# Mass spectrometric proteome profiling using a deep spectral library reveals homogenization of right and left atrial proteomes in persistent atrial fibrillation patients

**DOI:** 10.1093/cvr/cvag076

**Published:** 2026-04-02

**Authors:** Aiste Liutkute, Takiy-Eddine Berrandou, Stefanie Kestel, Moritz Schnelle, Olga Dschun, Hugo Alejandro Amedei, Lisa Neuenroth, Eric Rytkin, Oksana Kyshynska, George Kensah, Aschraf El-Essawi, Ahmad Fawad Jebran, Bernhard C Danner, Hassina Baraki, Ingo Kutschka, Felix Bremmer, Henning Urlaub, Constanze Schmidt, Nabila Bouatia-Naji, Igor R Efimov, Bianca J J M Brundel, Christof Lenz, Niels Voigt

**Affiliations:** Institute of Pharmacology and Toxicology, University Medical Center Göttingen, Germany; DZHK (German Center for Cardiovascular Research), Partner Site Lower Saxony, Germany; Cluster of Excellence ‘Multiscale Bioimaging: From Molecular Machines to Networks of Excitable Cells’ (MBExC), University Medical Center Göttingen, Robert-Koch-Str. 40, 37075 Göttingen, Germany; Université Paris Cité, INSERM, PARCC, Paris, France; Institute of Pharmacology and Toxicology, University Medical Center Göttingen, Germany; DZHK (German Center for Cardiovascular Research), Partner Site Lower Saxony, Germany; Department of Clinical Chemistry, University Medical Center Göttingen, Germany; Institute of Pathology, University Medical Center Göttingen, Germany; Department of Clinical Chemistry, University Medical Center Göttingen, Germany; Department of Clinical Chemistry, University Medical Center Göttingen, Germany; Bioanalytical Mass Spectrometry Group, Max Planck Institute for Multidisciplinary Sciences, Germany; Department of Biomedical Engineering, Northwestern University, Chicago, IL, USA; Department of Thoracic and Cardiovascular Surgery, University Medical Center Göttingen, Germany; Department of Thoracic and Cardiovascular Surgery, University Medical Center Göttingen, Germany; Department of Thoracic and Cardiovascular Surgery, University Medical Center Göttingen, Germany; Department of Thoracic and Cardiovascular Surgery, University Medical Center Göttingen, Germany; Department of Thoracic and Cardiovascular Surgery, University Medical Center Göttingen, Germany; Department of Thoracic and Cardiovascular Surgery, University Medical Center Göttingen, Germany; Department of Thoracic and Cardiovascular Surgery, University Medical Center Göttingen, Germany; Institute of Pathology, University Medical Center Göttingen, Germany; Cluster of Excellence ‘Multiscale Bioimaging: From Molecular Machines to Networks of Excitable Cells’ (MBExC), University Medical Center Göttingen, Robert-Koch-Str. 40, 37075 Göttingen, Germany; Department of Clinical Chemistry, University Medical Center Göttingen, Germany; Bioanalytical Mass Spectrometry Group, Max Planck Institute for Multidisciplinary Sciences, Germany; DZHK (German Center for Cardiovascular Research), Partner Site Lower Saxony, Germany; Department of Cardiology, University Hospital Heidelberg, Heidelberg, Germany; German Center for Cardiovascular Research Partner Site Heidelberg/Mannheim, Heidelberg University, Heidelberg, Germany; Department of Cardiology and Pneumology, University Medical Center Göttingen, Germany; Université Paris Cité, INSERM, PARCC, Paris, France; Department of Biomedical Engineering, Northwestern University, Chicago, IL, USA; Department of Biomedical Engineering, The George Washington University, Washington, DC, USA; Department of Medicine, Northwestern University, Chicago, IL, USA; Department of Physiology, Amsterdam UMC, Vrije Universiteit, Amsterdam Cardiovascular Sciences, Cardiomyopathy and Arrhythmia, Amsterdam, The Netherlands; DZHK (German Center for Cardiovascular Research), Partner Site Lower Saxony, Germany; Cluster of Excellence ‘Multiscale Bioimaging: From Molecular Machines to Networks of Excitable Cells’ (MBExC), University Medical Center Göttingen, Robert-Koch-Str. 40, 37075 Göttingen, Germany; Department of Clinical Chemistry, University Medical Center Göttingen, Germany; Bioanalytical Mass Spectrometry Group, Max Planck Institute for Multidisciplinary Sciences, Germany; Institute of Pharmacology and Toxicology, University Medical Center Göttingen, Germany; DZHK (German Center for Cardiovascular Research), Partner Site Lower Saxony, Germany; Cluster of Excellence ‘Multiscale Bioimaging: From Molecular Machines to Networks of Excitable Cells’ (MBExC), University Medical Center Göttingen, Robert-Koch-Str. 40, 37075 Göttingen, Germany

**Keywords:** Atrial fibrillation, Spectral library, Proteome, DIA-MS, Right atrium

## Abstract

**Aims:**

Pulmonary veins in the left atrium (LA) are well-established as a critical site for the initiation of atrial fibrillation (AF). Emerging evidence suggests that in persistent AF (persAF), AF triggers may extend beyond LA. However, the extent to which AF-associated remodelling involves the right atrium (RA) in persAF remains a subject of debate. To address this, we employed a proteomic approach aiming at investigating AF-associated remodelling in the RA relative to the LA in persAF.

**Methods and results:**

RA and LA samples were obtained from sinus rhythm (SR) patients, patients with persAF undergoing open-heart surgery, and non-failing donor hearts rejected for transplantation. A reference spectral library representing the human cardiac proteome was employed to assess the RA and LA proteomes by data-independent acquisition mass spectrometry. Protein levels were quantified by immunoblotting of human atrial tissue. Plasma levels of NT-proANP and NT-proBNP were measured in SR and persAF patients. Fibrosis levels were quantified in paraffin-embedded sections using Masson-Goldner trichrome staining. A spectral library representing 13 539 human proteins was generated from five anatomical regions of five independent donor hearts. In persAF RA, we observed marked myolysis, excessive extracellular matrix deposition and a prominent similarity to the failing ventricular proteome, all comparable to persAF LA. Although significant proteomic differences were observed between the RA and LA from SR patients, a comparison of RA and LA proteomes in persAF patients revealed proteome homogenization between the two atrial chambers. RA contributes to this homogenization by losing RA-specific markers, while gaining LA-specific markers.

**Conclusion:**

Our findings suggest that RA undergoes comparable AF-associated remodelling to LA, contributing to atrial proteome unification which represents a hallmark of persAF.

## Graphical Abstract

**Graphical Abstract:**
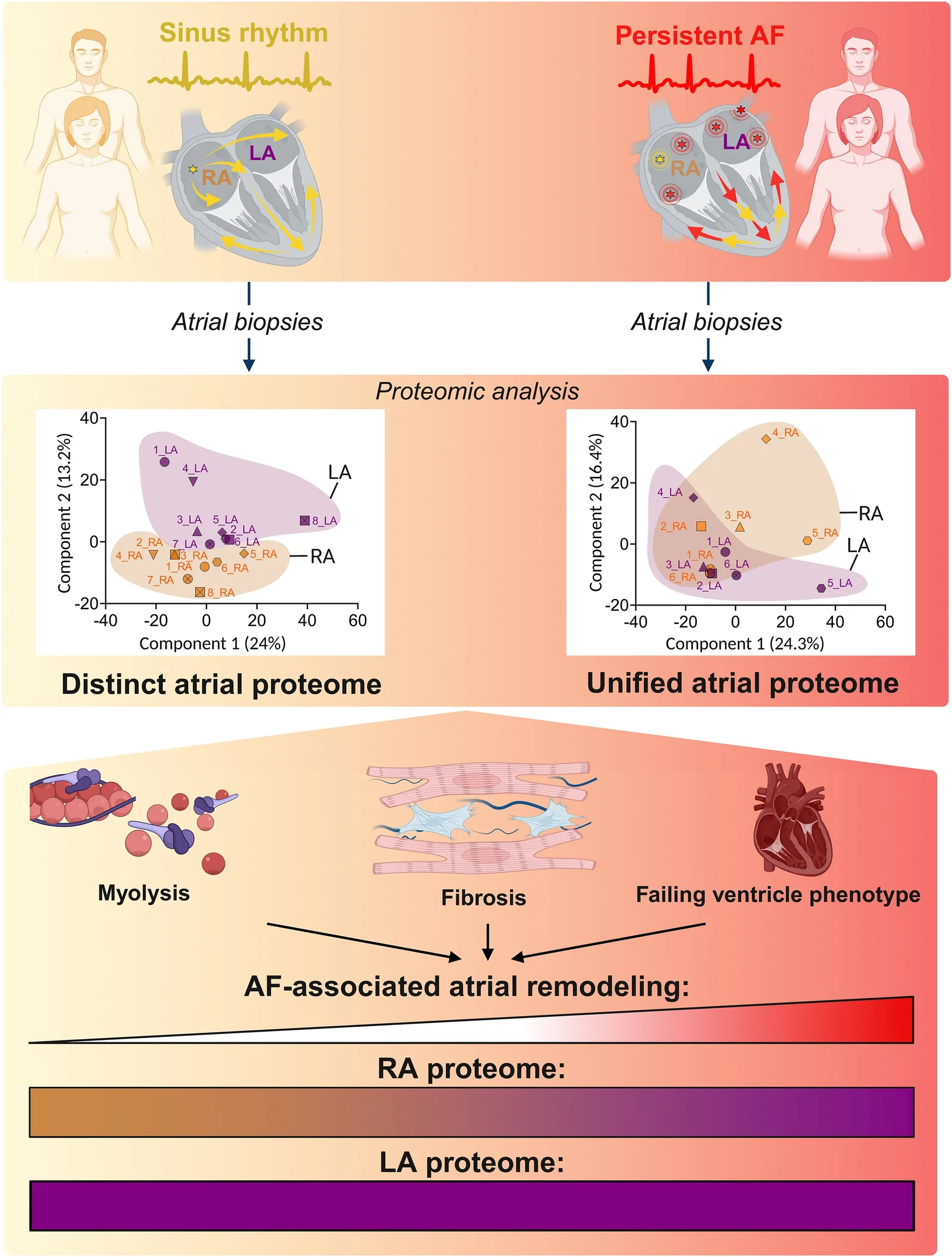

**Time of primary review: 45 days**

**See the editorial comment for this article ‘Proteomic insights into bi-atrial remodelling in persistent atrial fibrillation’, by J. Barallobre-Barreiro and M. Mayr, https://doi.org/10.1093/cvr/cvag104.**

## 1. Introduction

Atrial fibrillation (AF) is the most common cardiac arrhythmia associated with increased mortality and morbidity.^1,2^ Against the background of an aging population, the incidence of AF will further increase and will create a substantial socio-economic burden. Despite tremendous efforts in identifying underlying mechanisms during the last years, there is a lack of effective therapeutic strategies, particularly for patients with long-term persistent AF (persAF).^3^

AF is a chronic progressive disease. Patients often initially show paroxysmal AF (pAF), consisting of self-terminating episodes lasting ≤ 7 days, then persistent and finally long-lasting persistent states that fail to self-terminate.^1,2^ The progressive nature of AF is thought to result from atrial remodelling leading to structural, electrophysiological, Ca^2+^-handling, and metabolic abnormalities that facilitate the initiation and maintenance of the arrhythmia.^4,5^

In pAF, high-frequency re-entrant sources (rotors) in the left atria (LA) and pulmonary veins (PV) play an important role in the initiation and maintenance of the arrhythmia. These dominant high-frequency sources in the LA create left-to-right atrial frequency gradients that were previously shown in sheep model^6^ and in patients with pAF.^7,8^ Accordingly, ablation of LA dominant frequency sites and PV has been shown to terminate pAF, supporting the notion that LA sites ‘drive’ AF.

However, LA-to-right atrium (RA)-dominant frequency gradients are clearer in patients with pAF than those with persAF,^8^ where the efficacy of PV ablation is significantly reduced.^1^ This diminished effectiveness may be attributed to the increased AF-associated remodelling in persAF, which stabilizes re-entrant circuits.^9,10^ Despite this, strategies targeting atrial remodelling have so far been unsuccessful in maintaining sinus rhythm (SR).^9,11^ In addition, it has been suggested that there may also be high-frequency sources in the RA, which may promote AF maintenance in persAF, indicating a more prominent role of the RA in AF progression.^8,12^ This suggests a potential role for RA remodelling in the pathophysiological mechanisms underlying persAF.

In SR there is a clear difference in gene expression and ion channel profile between right and left atria.^12,13^ Transcription factor paired-like homeodomain 2 (PITX2) has been suggested to play a significant role in the left-right asymmetry by modulating gene expression, ensuring the proper development and functional differentiation of the LA.^14^ Despite these apparent differences and the importance of RA in AF pathophysiology, it is largely unknown whether AF-associated remodelling is different between the RA and LA.

Here, we established a proteomic library of the human cardiac proteome in order to assess the RA and LA proteomes by data-independent acquisition mass spectrometry (DIA-MS), aiming to identify distinct protein abundance patterns and molecular markers that contribute to the functional and structural differences between the atria. Furthermore, we analyzed (and compared) left and right atrial samples from patients with persAF to uncover potential pathways involved in AF maintenance in a chamber-specific manner, highlighting the differences between the RA and LA.

## 2. Methods

A detailed methods description is provided in the Supplementary material online, Material.

This study was approved by the George Washington University Institutional Review Board and the ethics committee of Göttingen University (No. 4/11/18) and conducted following the Declaration of Helsinki.

### 2.1 Data availability

The MS proteome data have been deposited to the ProteomeXchange Consortium via the PRIDE^15^ partner repository with the dataset identifier PXD055180. The analyzed and processed data supporting the findings of this study are provided in the Supplementary Data files, with a detailed description of their contents available in Supplementary material online, *Table S1*.

### 2.2 Human tissue samples

**Figure 1:**
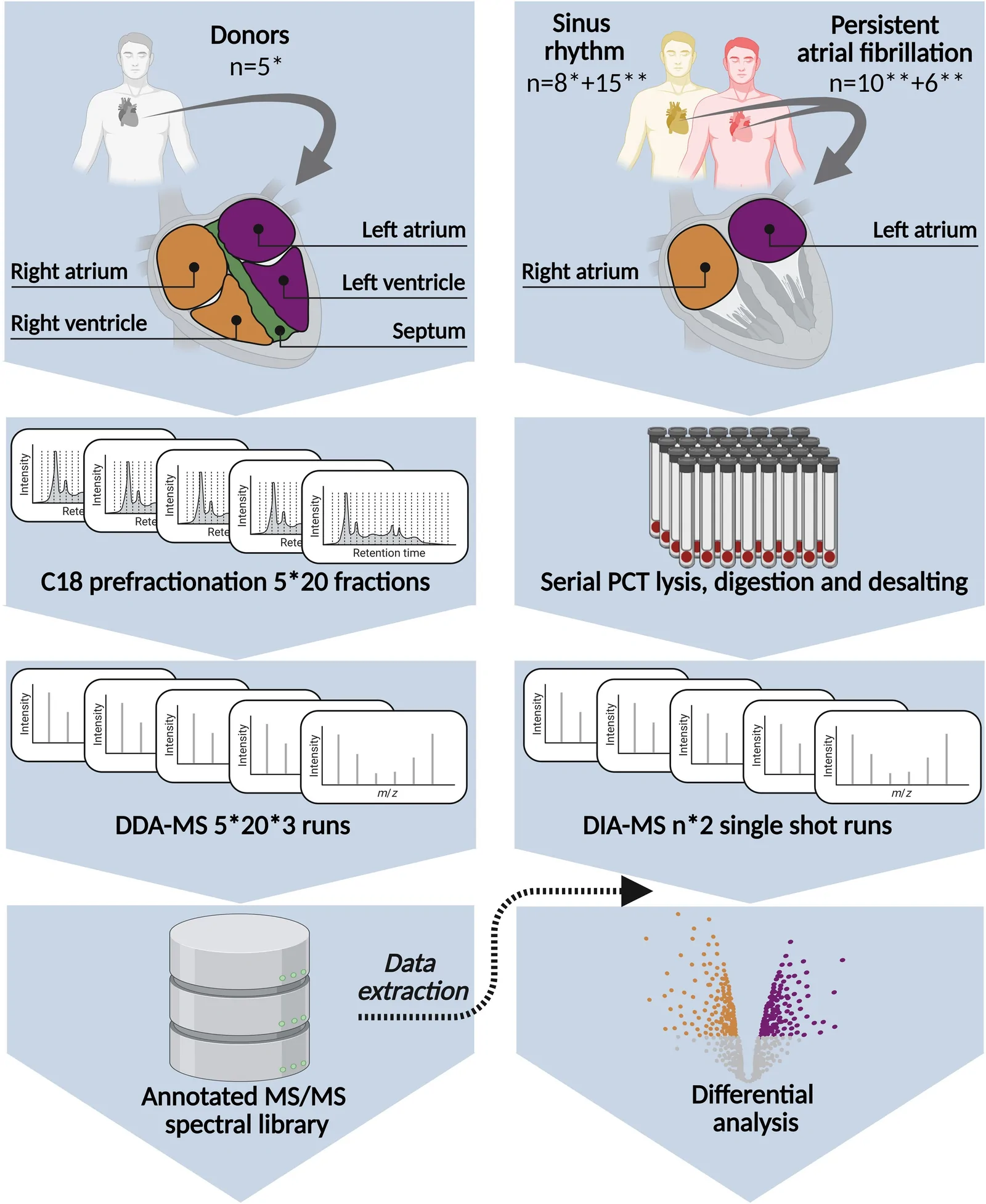
Experimental setup and workflow. The pipeline for generating an MS/MS reference spectral library including the isolation of selected anatomical regions from the donor hearts that were subjected to a DDA-MS (left). Right and left atrial appendage samples were collected from patients with SR and persAF and analyzed via DIA-MS (right). Application of the MS/MS spectral library enabled a robust analysis of the AF proteome. *Patients included in the cohort summarized in Supplementary material online, *Table S2*, **Patients included in the cohort summarized in *Table S3*. Numbers indicate a total number of patients. DDA, data-dependent acquisition; DIA, data-independent acquisition; MS, mass spectrometry; PCT, pressure cycling technology. The figure was created with BioRender.com.

Of the 8 deidentified, explanted non-failing human hearts from deceased donors that were rejected for transplantation and included in this study, 5 were used for spectral library generation (see Supplementary material online, *Table S2*, *Figure 1*). All hearts were explanted by a cardiothoracic surgeon and rapidly transferred to a research team member in the operating room. Coronary artery perfusion with ice-cold cardioplegic solution (110 mM NaCl, 1.2 mM CaCl_2_, 16 mM KCl, 16 mM MgCl_2_, and 10 mM NaHCO_3_) was used to arrest hearts. Hearts were then packed in a cooler with a cold cardioplegic solution to transport to the laboratory, with a total time from heart excision to laboratory of ∼20 min. Samples for proteomic analysis were dissected in a cardioplegic solution and snap-frozen in liquid nitrogen.

RA samples were obtained during open-heart surgery from 15 SR patients and 16 patients in long-standing persistent AF who were in AF during tissue sampling (see Supplementary material online, *Table S3*, *Figure 1*). In 6 patients with persAF, paired LA samples were available and were used for intra-patient RA vs. LA comparisons. For the corresponding control comparisons, RA and LA samples from the 8 aforementioned non-failing donor hearts were employed.

Immunoblot experiments comparing RA-SR vs. RA-persAF were carried out in an independent patient cohort comprising 28 and 24 patients with SR and persAF, respectively (see Supplementary material online, *Table S4*).

Histology experiments comparing RA-SR vs. RA-persAF and LA-persAF were carried out in an independent patient cohort comprising 13 and 6 patients with SR and persAF, respectively (see Supplementary material online, *Table S5*).

Each surgery patient gave a written informed consent. Excised appendages were snap-frozen in liquid nitrogen for biochemical, histological and mass spectrometry (MS) studies.

### 2.3 Proteome profiling: pressure cycling tissue lysis and protein digestion

All tissue samples were run on the same LC-MS instrument using the same chromatography separation method. Tissue samples were mixed with 30 µL lysis buffer (8 M urea in 100 mM aqueous NH_4_HCO_3_ with pH 8.0), a complete protease inhibitor cocktail (Roche, Penzberg, DE) and PhosSTOP phosphatase inhibitor cocktail (Roche) in PCT microtubes (Pressure Biosciences, Easton/MA, U.S.A), followed by 1 min centrifugation at 5000*×g*. Following 3 × 10 s sonication cycles microtubes were sealed with micropestles. Lysis was achieved in a Barocycler 2320XT pressure cycler (Pressure Biosciences) by 60 pressure cycles consisting of 50 s at 45 kpsi, followed by 10 s at normal pressure.^16^ Next, samples were reduced and alkylated in a shaker at 600 rpm, using 5 mM of tris(2-carboxyethyl) phosphine and 20 mM iodoacetamide for 30 min at 30°C, diluted to urea concentration of 1.6 M and trypsinated (Promega, 1:20 enzyme-to-substrate ratio) using pressure cycling (90 cycles, 50 s at 20 kpsi followed by 10 s at normal pressure, 33°C). The processed samples were acidified to 1% trifluoroacetic acid, desalted on reversed phase C18, taken to dryness in a SpeedVac and stored at −20°C until further use.^16^

### 2.4 Proteome profiling: mass spectrometry

Prior to MS measurement, samples were reconstituted in 0.1% trifluoroacetic acid and 2% acetonitrile containing indexed retention time standard peptides (Biognosys, Schlieren, Switzerland), which, in combination with dynamic recalibration in the Spectronaut software, allows for correction of longitudinal retention time shifts and ensures accurate precursor matching between the library and study samples. For generation of a peptide library, 150 µg of peptide were pooled for each cavity including LA, RA, left ventricle (LV), right ventricle (RV) and septum, and separated into twenty fractions using a basic pH reversed phase C18 separation (*Figure 1*) on a fast protein liquid chromatography system (äkta pure, Cytiva, Marlborough/MA, USA) and a staggered pooling scheme.

400 ng equivalents of digested protein were analyzed on a nanoﬂow chromatography system (Dionex Ultimate nanoRSLC, Thermo Fisher) coupled to a hybrid timed ion mobility-quadrupole-time of flight mass spectrometer (timsTOF Pro 2, Bruker, Bremen, DE) as previously described.^17^

Data-dependent analysis (DDA) of the fractionated cavity-specific pools was performed in PASEF mode^18^ (*Figure 1*), with ten scans per topN acquisition cycle as previous described.^17^ Three technical replicates (i.e. MS injection replicates) per C18 fraction were acquired.

Data-independent analysis (DIA) of individual cardiac biopsies was performed in diaPASEF mode^19^ using a 100 min linear gradient and a customized 20 × 2 variable window size acquisition method covering ranges of *m/z* 350–1350 and 1/K_0_ = 0.7–1.5 Vs cm^−2^, respectively, generated with the pyDIAid script^20^ (*Figure 1*). Two technical replicates per sample were acquired.

### 2.5 Biochemical studies

Protein abundance was quantified by immunoblot as previously described^21,22^, and normalized to calsequestrin (see Supplementary material online, *Table S6*).

### 2.6 Histology

The samples were fixed in 10% formalin and embedded in paraffin. Paraffin-embedded tissue blocks were cooled and sectioned at 2 µm thickness using a sliding microtome (Leica SM2000R; Leica Biosystems, Germany). Sections were transferred to a water bath at room temperature, mounted onto glass slides, flattened in a stretching bath and air-dried before staining.

Masson-Goldner trichrome staining was performed on an automated stainer (Tissue-Tek Prisma E2S; Sakura Finetek, Germany) with integrated deparaffinization in xylene and rehydration through a graded ethanol series, followed by sequential incubation in iron haematoxylin, three Goldner solutions, acetic acid, and completed with dehydration in ethanol as well as clearing in xylene.

Stained sections were mounted using an automated coverslipper (Tissue-Tek Film 4740; Sakura Finetek, Germany) with a coverslipping film. Whole-slide images were acquired using an EVOS FL Auto 2 imaging system (Thermo Fisher) with a 10× objective. Quantification of intramuscular fibrosis (fibrotic area/total tissue area) was performed using ImageJ software.

### 2.7 Plasma NT-proBNP and NT-proANP measurements

Blood samples were obtained during open-heart surgery via a central venous catheter from a different patient cohort (see Supplementary material online, *Table S7*). Following centrifugation at 2700 *×g* for 10 min, EDTA plasma samples were stored at −80°C until further analysis. For plasma quantifications of the N-terminal prohormone of brain natriuretic peptide (NT-proBNP) and atrial natriuretic peptide (NT-proANP), samples were thawed and measured using the “Elecsys proBNP II” electro-chemiluminescence immunoassay (ECLIA) (Roche Diagnostics, Germany) on the automated cobas e 801 (Roche Diagnostics) for NT-proBNP, and the “proANP (1–98)” enzyme-linked immunoassay (ELISA) (Biomedica, Austria) on the automated Dynex DSX (Dynex Technologies, USA) for NT-proANP. Both methods were performed according to the respective manufacturers’ instructions and fulfill highest quality standards as they are used routinely in patient care at the central laboratory of the University Medical Centre Göttingen (UMG).

### 2.8 Statistical and bioinformatics analysis

Extensive MS proteomics data, including sample-level variability and technical reproducibility, is available at the Supplementary Data  *S1*. The Generalized Estimating Equations (GEE) model was applied using the ‘geepack’ R package for all comparisons of log_2_-transformed protein data.^23,24^  *P* values were adjusted for multiple testing using the Benjamini–Hochberg method^25^ via the ‘p.adjust’ function from the ‘stats’ package in R. Depending on the analysis, the dataset was pre-filtered based on indicated threshold and processed in Perseus v.1.6.15.0^26^ for principal component analysis (PCA), Enrichr^27^ for enrichment analysis (Gene Ontology (GO), Kyoto Encyclopedia of Genes and Genomes (KEGG)^28^ and Molecular Signatures Database (MSigDB) Hallmark 2020,^29^), and Cytoscape^30^ for protein-protein interaction networks.

To summarize medication burden, we constructed *Drugs-PC1* as the first principal component of eight binary drug-use indicators (Digitalis, ACE inhibitors, AT1 blockers, β-blockers, dihydropyridines, diuretics, nitrates, lipid-lowering drugs) after centering and scaling.

In addition to the primary (unadjusted) GEE, we refit models including covariates age, sex, Drugs-PC1, coronary artery disease (CAD), and mitral/aortic valve disease (MVD/AVD). Discovery thresholds and correction were unchanged (FDR < 0.05 together with |log_2_FC| > 0.58).

Plasma NT-proANP and NT-proBNP concentrations were analyzed with linear regression on log_2_-transformed values, contrasting persAF vs. SR. An adjusted specification included age, sex, CAD, valve disease, and diuretic use. Because only two biomarkers were tested, we report two-sided nominal *P*-values without multiplicity correction. Immunoblot data with sample size *n* ≥ 10 were tested for normality using the Shapiro-Wilk normality test. For datasets that followed a normal distribution, comparisons were performed using the unpaired two-tailed Student’s *t*-test. Datasets with unequal variance were tested using the Welch's *t* test. For immunoblot data with sample size *n* < 10, comparisons were conducted using the Mann–Whitney *U* test. A *P* value < 0.05 was considered statistically significant.

## 3. Results

### 3.1 PersAF shows canonical AF markers

**Figure 2:**
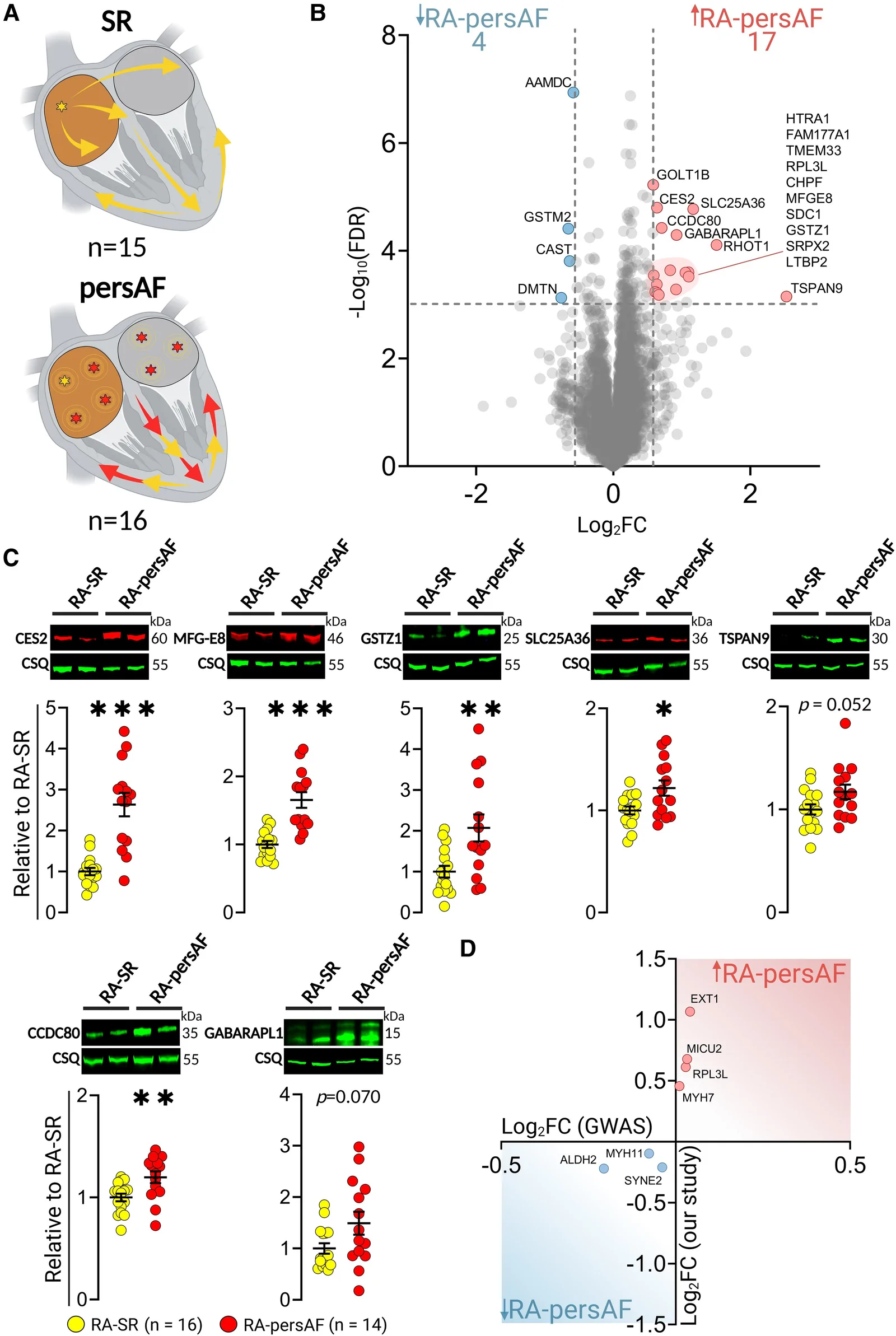
Canonical AF markers in RA-persAF proteome. (*A*) Schematic representation of conditions and sample locations. Numbers indicate number of patients. (*B*) Differential expression analysis comparing RA proteome from SR and persAF. Colours are assigned for the cases with FDR < 0.05 and |log_2_(FC)| > 0.58, other points are shown in grey. Legends show the total number of DEPs across both groups. (*C*) Immunoblots (top) and quantification (bottom) validating key dysregulated proteins in RA-persAF identified by proteomic analysis, including CES2, MFG-E8, GSTZ1, SLC25A36, TSPAN9, CCDC80, GABARAPL1. Data was normalized to CSQ (calsequestrin). * *P* < 0.05, ** *P* < 0.01, *** *P* < 0.001 vs. RA-SR. (*D*) Scatter plot showing positively correlating log_2_FC between RA-persAF markers and mapped genes associated with AF (published NHGRI-EBI catalog of GWAS). Pink colour represents up-regulation and blue colour represents down-regulation in persAF relative to SR. Statistical significance was determined using GEE analysis with Benjamini–Hochberg correction. FC, fold change; RA-persAF, right atrium from patients with persistent AF. The figure was created with BioRender.com.

To assess the proteomic profile associated with persAF, we compared RA samples obtained from patients with SR (RA-SR; *n* = 15) and RA samples from patients with persAF (RA-persAF; *n* = 16; Supplementary material online, *Table S3*). Our analysis identified 21 differentially expressed proteins [FDR < 0.05 and |log_2_(FC)| > 0.58] in RA-persAF samples, of which 17 were up-regulated and 4 were down-regulated (*Figure 2B*, Supplementary Data  *S2*). Because age, sex, cardiovascular comorbidity and medication can influence atrial proteomes, we evaluated potential confounding. Baseline age and sex did not differ significantly between groups (see Supplementary material online, *Table S3*), and medication use across eight major drug classes was similar (see Supplementary material online, Figure  *S1A*). Sensitivity analyses confirmed robustness: adjusting for overall medication burden (Drugs-PC1) yielded near-identical group-effect estimates (*R*^2^ = 0.969; RMSE = 0.034; Supplementary material online, Figure  *S1B*), and a fully adjusted model (Age, Sex, Drugs-PC1, CAD, MVD/AVD) produced tightly aligned effects (*r* = 0.917; RMSE = 0.082), with 16 proteins meeting discovery thresholds; nine overlapped the primary set and all shared the same direction of effect (see Supplementary material online, Figure  *S1C*; Supplementary material online, *Table S8*). Taken together, the persAF-associated proteomic signal is consistent and robust to demographic, clinical, and medication adjustment.

To support the reliability of the proteomic findings, we performed immunoblot validation in an independent patient cohort (SR, *n* = 16; persAF, *n* = 14). Supplementary material online, *Table S4* lists the clinical characteristics of all patients used for immunoblotting (SR, *n* = 28; persAF, *n* = 24), including those for additional blots not shown in *Figure 2C*. Among the selected key proteins (CES2, MFG-E8, GSTZ1, SLC25A36, TSPAN9, CCDC80, and GABARAPL1) the abundance patterns were found to be consistent with the proteomic results (*Figure 2C*). These included statistically significant differences for most proteins, while others showed trends toward significance, all with regulation in the same direction as observed in the proteomic dataset.

To further expand the probability of identifying proteins with a potential association to persAF, we intersected over-represented proteins from our dataset with gene variants associated with AF from the genome-wide association study (GWAS) catalog (*Figure 2C*, Supplementary Data  *S3*). The analysis highlighted the muscle function regulator RPL3L as one of the most significantly associated proteins across both datasets (GWAS *P* = 2.0E−14, our *P* = 5.8E−4). Other positively correlating candidate proteins include EXT1, which is involved in extracellular matrix (ECM) organization; a key regulator of mitochondrial calcium uniporter MICU2; and myosin heavy chain slow isoform MYH7. Conversely, negatively correlating proteins include a key metabolic enzyme involved in the detoxification of aldehydes ALDH2; a smooth muscle isoform of myosin heavy chain MYH11; and a component of the linker of nucleoskeleton and cytoskeleton complex SYNE2. Although we are unable to rule out whether the alterations in the aforementioned proteins in our patient cohort are due to genetic variants or due to the maintenance of AF, the overlap with AF-related genes suggests that these proteins are more likely to be related to AF and, therefore, should be prioritized in future AF studies.

### 3.2 PersAF shows canonical AF pathophysiological pathways

**Figure 3:**
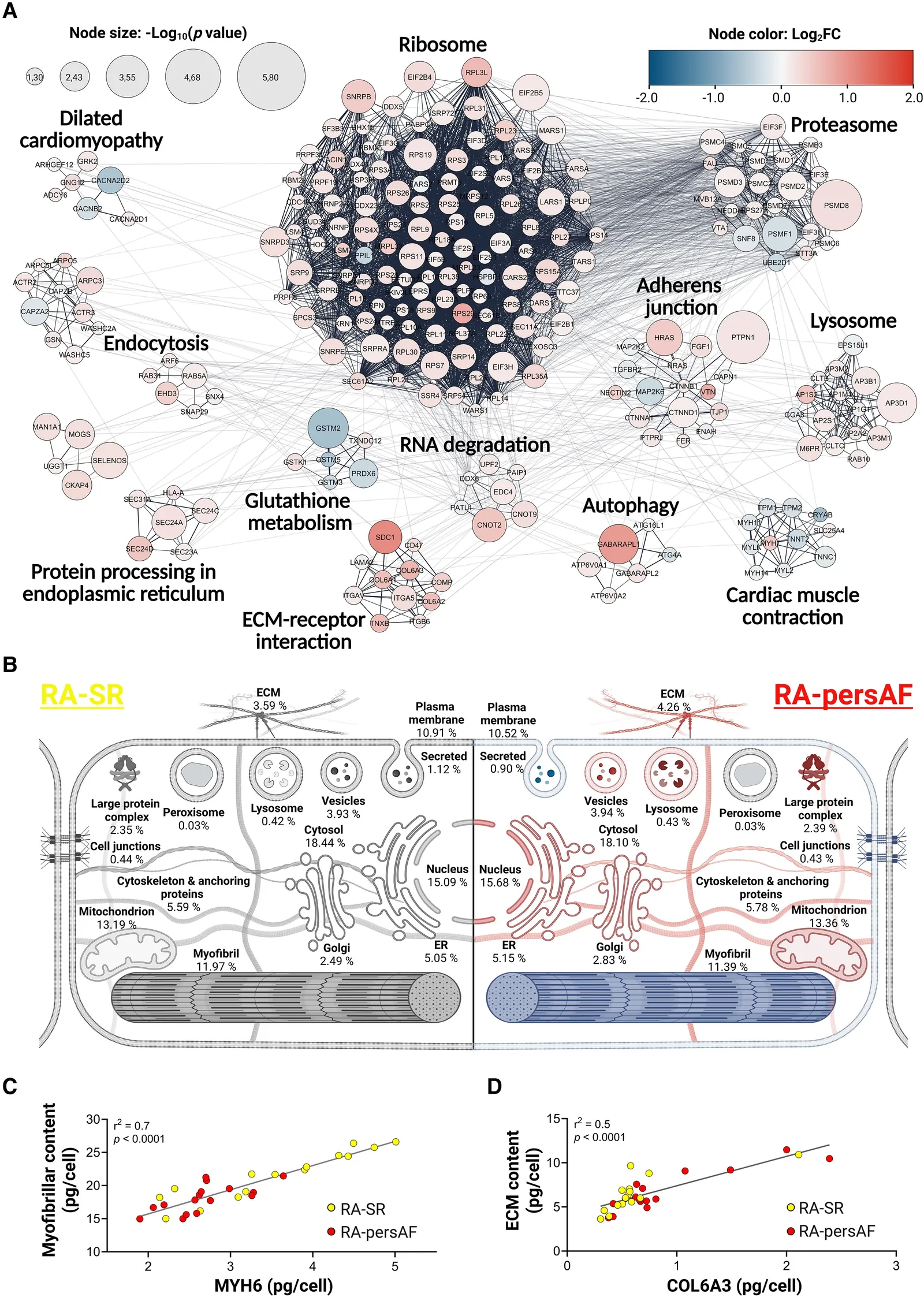
Protein association network and subcellular proteome model of RA-persAF. (*A*) Protein association network with respective pathway enrichment in RA-AF proteome. For the full-size version, see Supplementary material online, Figure  *S3*. Proteins are shown in nodes, node size represents −log_10_  *p* value, colour represents log_2_ FC and interaction intensity reflects confidence level. For each cluster, KEGG pathway associations were retrieved. Statistical significance was determined using GEE analysis with Benjamini–Hochberg correction. ECM, extracellular matrix; FC, fold change. (*B*) Schematic representation of an average right atrial appendage cardiomyocyte illustrating the subcellular distribution of protein mass fractions per cell, estimated using proteomic ruler approach. Values are normalized to total cellular protein content. Left: RA-SR (control). Right: RA-persAF. In both panels, compartments are labelled with their absolute percentage contribution (%) to total protein mass per cell, enabling direct comparison between conditions. (*C*) Spearman's correlation scatter plot showing the relationship between MYH6, a driver of myofibrillar content change in persAF, and myofibrillar content in RA samples from SR and persAF patients. (*D*) Spearman's correlation scatter plot showing the relationship between COL6A3, a driver of ECM content change in persAF, and ECM content in RA samples from SR and persAF patients. Diagonal line in scatter plots: global (shared) non-linear fit. ECM, extracellular matrix; ER, endoplasmic reticulum; FC, fold change; RA-persAF, right atrium from patients with persAF; RA-SR, right atrium from patients with sinus rhythm. The figure was created with BioRender.com.

Next, the uniquely regulated pathways in RA-persAF compared with controls were assessed using KEGG pathway analysis. The largest up-regulated cluster is related to the ribosome pathway (adj. *P* = 6.31 × 10^−20^; *Figure 3*, Supplementary Data  *S4*), indicating heightened protein synthesis in RA-persAF. This is additionally corroborated by the observation that, when the dataset is pre-filtered for positively abundant DEPs only, cytoplasmic translation remains the most up-regulated biological process (see Supplementary Data  *S4*) consistent with the ribosome cluster being the dominant up-regulated feature in RA-persAF.

This is also supported by two up-regulated clusters representing proteins involved in protein processing in the endoplasmic reticulum (ER). Correspondingly, the RA-persAF proteome appears enriched in proteasome proteins, implying enhanced protein degradation. This finding is corroborated by increased lysosome, autophagy, and RNA degradation-related proteins. The dense interaction between the ribosome and proteasome clusters potentially suggests a tight co-ordination between the two. Furthermore, a marked decrease in cardiac contractile proteins was observed, including TNNC1, CRYAB, TPM1, TPM2, MYLK, MYH11, MYH14, MYL2, and TNNT2. This interpretation is reinforced by the fact that, when the dataset is pre-filtered for negatively abundant DEPs only, cardiac muscle contraction emerges among the most down-regulated biological processes (see Supplementary Data  *S4*). This is in accordance with previous publications linking myolysis and enhanced protein degradation processes with AF.^31,32^

In addition to myolysis, we observed increased glycolysis in RA-persAF which may be another overlapping compensatory response with HF and is in line with previous AF proteomic studies^33^ (see Supplementary Data  *S5*).^34,35^

Finally, one of the most striking decreases was observed in glutathione metabolism, frequently indicative of increased oxidative stress, another well-established factor in AF.^36^ In contrast, the cluster of ECM and receptor interaction demonstrated one of the most pronounced increases with robust enrichment in collagens and integrins. Proteins involved in ECM and receptor interaction appeared notably interconnected with up-regulated adhesion junction proteins, suggesting a potential crosstalk between the two pathways.

### 3.3 Subcellular compartmentalization is altered in RA-persAF: ECM and myolysis

To further link the enriched biological pathways with their respective subcellular compartment, we employed the proteomic ruler approach.^37,38^ Briefly, this approach uses the total MS signal from histones as an internal reference to convert MS intensity into protein mass. Since both DNA and histones are present in known and consistent amounts per diploid nucleus, the histone signal serves as a reliable proxy for estimating total protein content per cell as well as the amount of each protein available in the dataset. Additionally, annotating each protein by its predominant subcellular localization enables the estimation of how cellular compartments may be affected relative to the observed proteomic changes.

In RA from the patients with SR, the nucleus, mitochondria and myofibrils were the principal contributors to the total protein mass, accounting for 15, 13 and 12%, respectively (*Figure 3B*, Supplementary material online, Figure  *S3*, Supplementary Data  *S6*).

In RA-persAF, the most notable increase was in ECM proteins along with increased protein synthesis compartments, including ER and Golgi. In line with KEGG pathways/clusters, a notable loss in myofibrillar protein mass was observed in persAF. MYH6 and COL6A3 were the most altered myofibrillar and ECM proteins in RA-persAF, respectively (see Supplementary Data  *S6*). This is illustrated by a strong correlation between the myofibrillar compartment and MYH6, as well as between the ECM compartment and COL6A3, in SR and persAF (*Figure 3C,D*).

Interestingly, the total protein mass per average cell was lower in RA-persAF (0.158 ng) compared with RA-SR (0.180 ng; Supplementary Data  *S6*). As atrial cardiomyocytes tend to be larger in AF,^22^ this reduction could potentially be due to the overrepresentation of non-cardiomyocyte populations in RA-persAF, as previously observed in LA from patients with persAF.^39^ Note that smaller cells contribute approximately equally to histone signal but less to total protein signal, altering their ratio and thus reducing the total protein mass. In addition, <20% of human adult atrial cardiomyocytes are expected to be non-mononucleated,^40,41^ thus, compartment mass estimates should be interpreted with caution. Nevertheless, an analogous analysis performed without normalization to histone signal yielded highly comparable results (see Supplementary material online, Figure  *S4*), suggesting that histone-based scaling is unlikely to be a major factor underlying the observed compartmental protein mass distributions.

### 3.4 RA-persAF exhibits failing ventricle-like phenotype

Previous studies have demonstrated a shift in myosin isoform abundance in persAF, marked by a reduction in MYH6 and an up-regulation of MYH7.^22,42,43^ This change may be interpreted as a potential ‘ventricularization’ of the atria. To investigate this phenomenon in RA-persAF proteome, we first derived a set of robust atrial and ventricular markers from two independent, publicly available cardiac proteomics datasets.^38,44^ Atrial and ventricular markers were identified in each dataset using two-sample *t*-tests with Benjamini–Hochberg correction and were further overlapped yielding high-confidence atrial (*n* = 53) and ventricular (*n* = 50) markers (see Supplementary material online, Figure  *S5*, top, Supplementary Data  *S7*). These shared markers were further validated using tissue-specific annotations from the TISSUES Experimental 2025 database,^45^ supporting their specificity and biological relevance (see Supplementary material online, Figure  *S5*, bottom).

**Figure 4:**
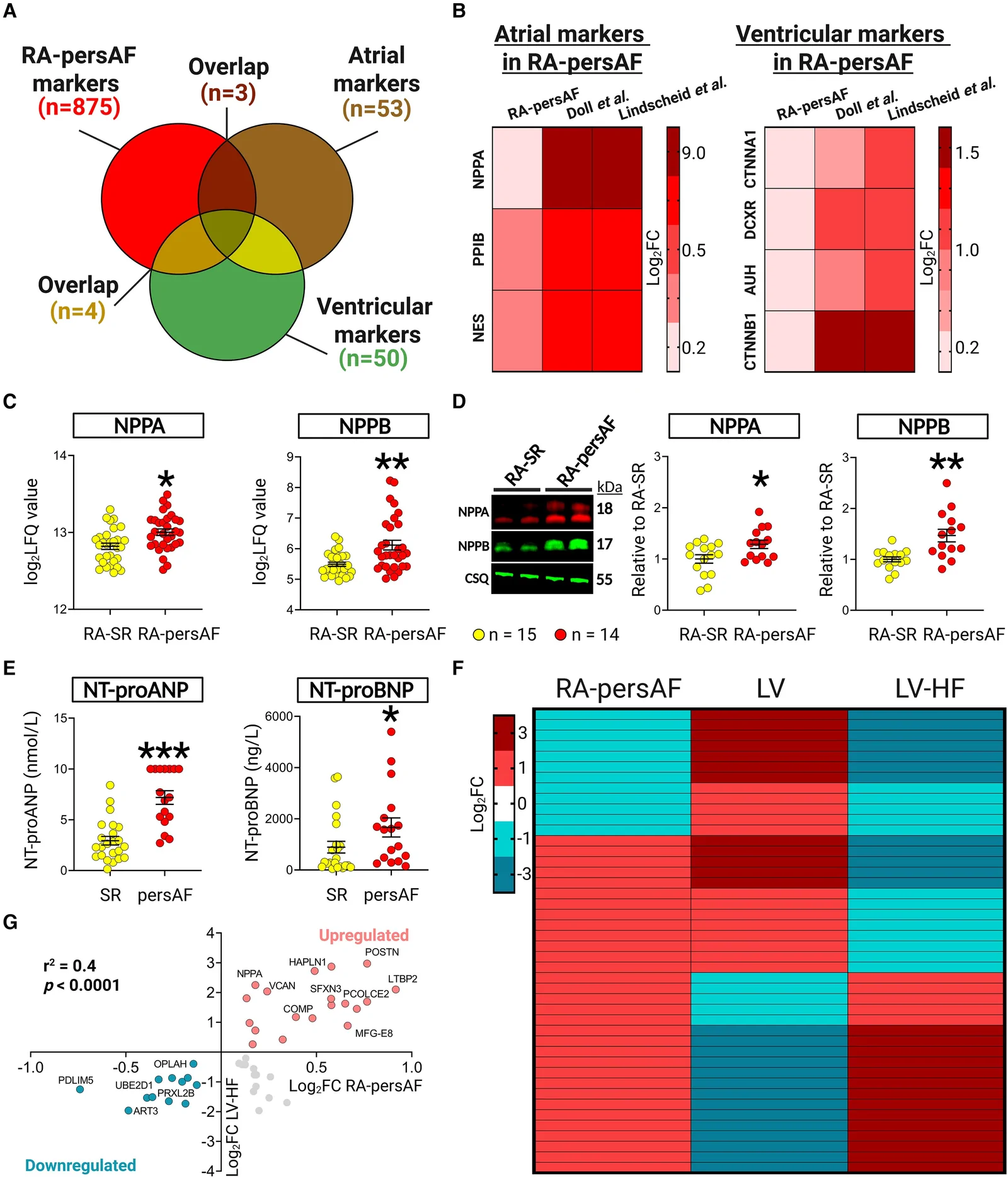
Profiling atrial and ventricular signature in RA-persAF proteome. (*A*) Venn diagram illustrating the overlap between RA-persAF markers (*n* = 875) and robust atrial (*n* = 53) and ventricular (*n* = 50) markers derived from two independent, publicly available cardiac proteomics datasets.^38,44^ (*B*) Heatmaps showing the log_2_FC of the overlapping atrial^38,44^ (left) and ventricular^38,44^ (right) markers across RA-persAF/RA-SR. (*C*) Comparison of NPPA (natriuretic peptide A) and NPPB (natriuretic peptide B) in RA-SR and RA-persAF, displayed as log_2_LFQ abundance (proteomics dataset). *P < 0.05, **P < 0.01 vs. RA-SR. (*D*) Immunoblots (left) and quantification (right) of NPPA and NPPB in RA-SR and RA-persAF, normalized to CSQ (calsequestrin). *P < 0.05, **P < 0.01 vs. RA-SR. (*E*) Comparison of atrial natriuretic peptide (NT-proANP) and brain natriuretic peptide (NT-proBNP) blood plasma levels in patients with SR (*n* = 23) and persAF (*n* = 17). *P < 0.05, ***P < 0.001 vs. SR. (*F*) Heatmap generated based on the log_2_FC of DEPs in RA-persAF/RA-SR (*P* < 0.05), LV/LV-HF and LV-HF/LV (*P* < 0.05). (*G*) Spearman's correlation scatter plot showing the distribution of DEPs from (*E*) based on their log_2_FC in RA-persAF/RA-SR and LV-HF/LV. Data are presented as mean ± SEM. Statistical significance for proteomics data was determined using GEE analysis with Benjamini–Hochberg correction, whereas comparison of immunoblots was made using the Student’s *t*-test with or without Welch correction. FC, fold change; LV, left ventricle; LV-HF, failing left ventricle; RA-SR, right atrium from patients with sinus rhythm; RA-persAF, right atrium from patients with persistent AF.

When RA-persAF markers (up-regulated relative to RA-SR) were overlapped with this validated set of chamber-specific markers, no clear shift toward either atrial or ventricular identity was observed in RA-persAF proteome. Instead, the overlap with chamber-specific markers was relatively balanced, suggesting the absence of global ventricularization in RA-persAF. Among the overlapping proteins, atrial markers included NPPA, PPIB and NES, while ventricular markers encompassed CTNNA1, DCXR, AUH, and CTNNB1 (*Figure 4B*, Supplementary Data  *S7*).

Interestingly, the DCM pathway that emerged in the RA-persAF proteome (relative to RA-SR; *Figure 3*), along with the up-regulation of other HF hallmarks NPPA and NPPB^46^ (*Figure 4C, D*), pointed to shared features with HF. Consistently, NT-proANP and NT-proBNP were also significantly up-regulated in plasma samples from persAF patients compared with SR patients (*Figure 4E*; Supplementary material online, *Table S7*). Plasma NT-proANP levels remained significantly elevated after adjusting for age, sex, CAD, valve disease, and diuretic use, whereas NT-proBNP levels showed a similar trend but did not reach statistical significance following adjustment.

To explore whether persAF exhibits broader similarities to HF (specifically DCM), we compared the proteome profile of atrial samples from persAF patients (relative to RA-SR; *P* < 0.05) with published proteome profile from failing human LV^47^ (LV-HF; *P* < 0.05; Supplementary Data  *S7*). The overlap of both pre-filtered datasets resulted in 44 proteins. Notably, the LV-HF group corresponds to the DCM cohort (relative to healthy donor) from Li *et al*., which was selected due to more severe systolic dysfunction and fewer confounding factors such as ischaemia and vascular comorbidities, enabling a more focused comparison of shared HF-related proteomic alterations. The LV-HF dataset from Li *et al*. was reprocessed and reanalyzed using our spectral library and the same GEE statistical approach applied to the RA-persAF vs. RA-SR dataset.

Using this exploratory framework, we observed a clustering between RA-persAF and LV-HF for the most of pre-filtered proteins (*Figure 4F*). This is further supported by Spearman’s correlation analysis indicating a positive relationship between the two datasets (*Figure 4G*). Overlapping down-regulated proteins were predominantly linked to the metabolism (GPT, GALK1, GALT, PRXL2B, OPLAH), protein degradation (UBE2D1) and cytoskeleton (PDLIM5). In contrast, overlapping up-regulated proteins were mainly associated with ECM (MFG-E8, COMP, PCOLCE2, TNXB, COL6A3, POSTN, DPT, VCAN, LTBP2, and HAPLN1).

### 3.5 RA and LA proteomes become more uniform in persAF

**Figure 5:**
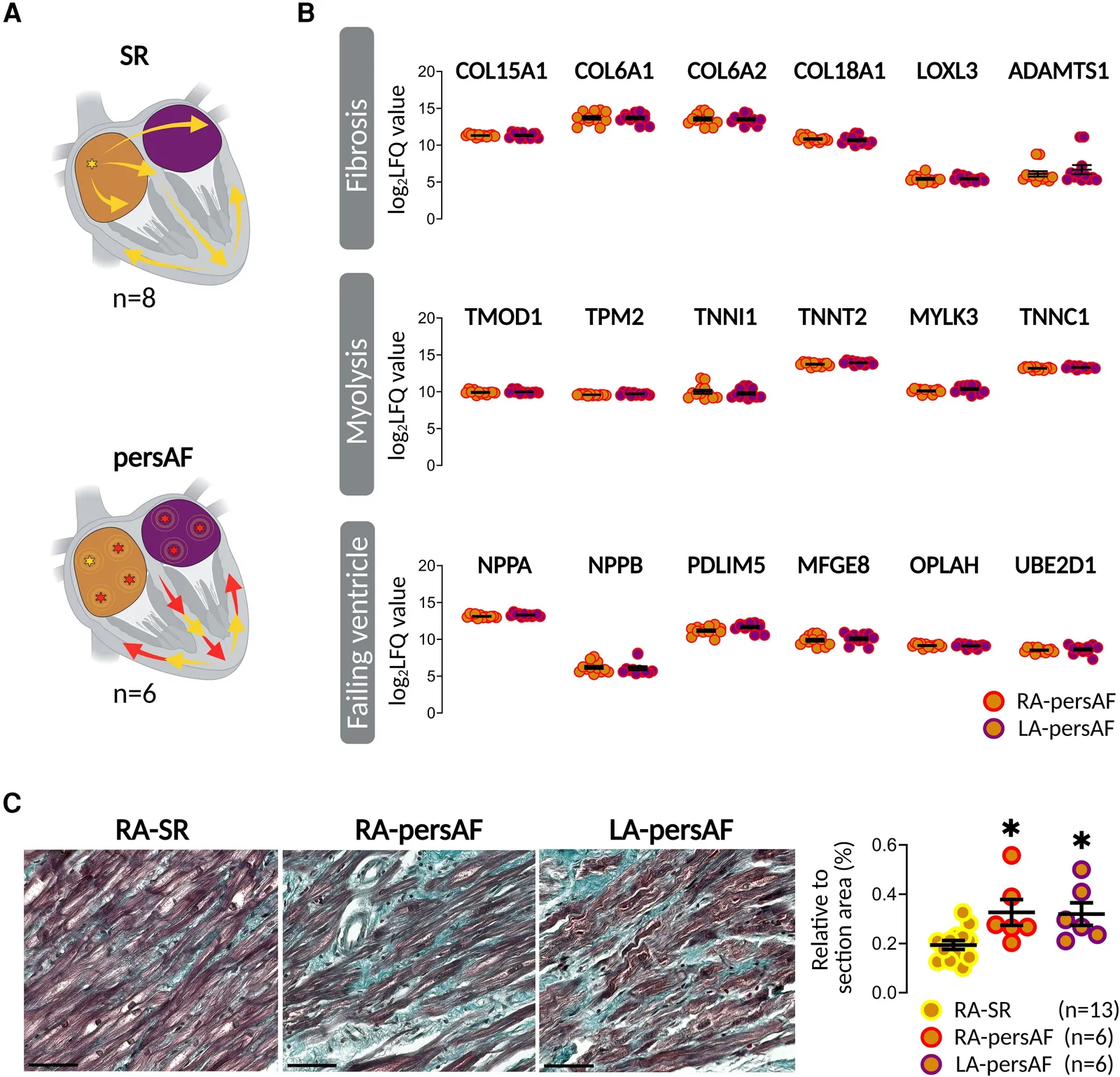
Comparative analysis of previously defined AF-associated remodelling features in RA- and LA-persAF. (*A*) Schematic representation of conditions and sample locations. Numbers indicate number of patients. (*B*) Quantitative comparison of representative fibrosis (top), myolysis (middle) and failing ventricle-like (bottom) proteins, displayed as log_2_LFQ abundance (proteomics dataset). Each dot represents a technical replicate/patient. (*C*) Representative Masson-Goldner trichrome-stained sections of the RA in SR (RA-SR), RA in persAF (RA-persAF), and LA in persAF (LA-persAF) patients (left) and Mean ± SEM of fibrotic area as a percentage relative to the total tissue section area (right). Scale bar: 50 µm. Each dot represents an individual patient. Statistical significance was assessed using the GEE analysis for proteomics data and Kruskal–Wallis test for histology data. * *P* < 0.05. Numbers indicate the number of patients. LFQ, label-free quantification.

To compare AF-associated proteome remodelling in RA and LA, we next analysed paired RA and LA samples from 8 SR and 6 persAF patients (*Figure 5A*). Among the 16 persAF patients from whom RA tissue was available for the analyses described above (see Supplementary material online, *Table S3*), paired LA tissue was available from 6 patients. For SR, RA-LA comparisons were made possible through a unique opportunity to use healthy donor hearts rejected from transplantation for non-cardiac reasons (see Supplementary material online, *Table S2*).

We first assessed the key features of AF-associated remodelling previously identified in our dataset (fibrosis, myolysis, and a failing ventricle-like proteomic profile; *Figures 3–5*) in both atria during persAF. Interestingly, the abundance of top proteins linked to these remodelling processes appeared to be similar in RA and LA samples from persAF patients (*Figure 5B*).

We further quantified atrial fibrosis relative to total tissue section in RA and LA using Masson-Goldner trichrome-stained paraffin sections from an independent patient cohort (see Supplementary material online, *Table S5*). This analysis revealed that both RA and LA from persAF patients exhibited significantly increased fibrosis compared with RA-SR controls, whereas no significant difference was observed between RA and LA within the persAF group (*Figure 5C*). After covariate adjustment (see Supplementary material online, *Table S5*), the direction of the effect was preserved, though statistical significance was not reached, likely reflecting the limited sample size and variance imbalance (13 vs. 6, F-test = 0.049).

**Figure 6:**
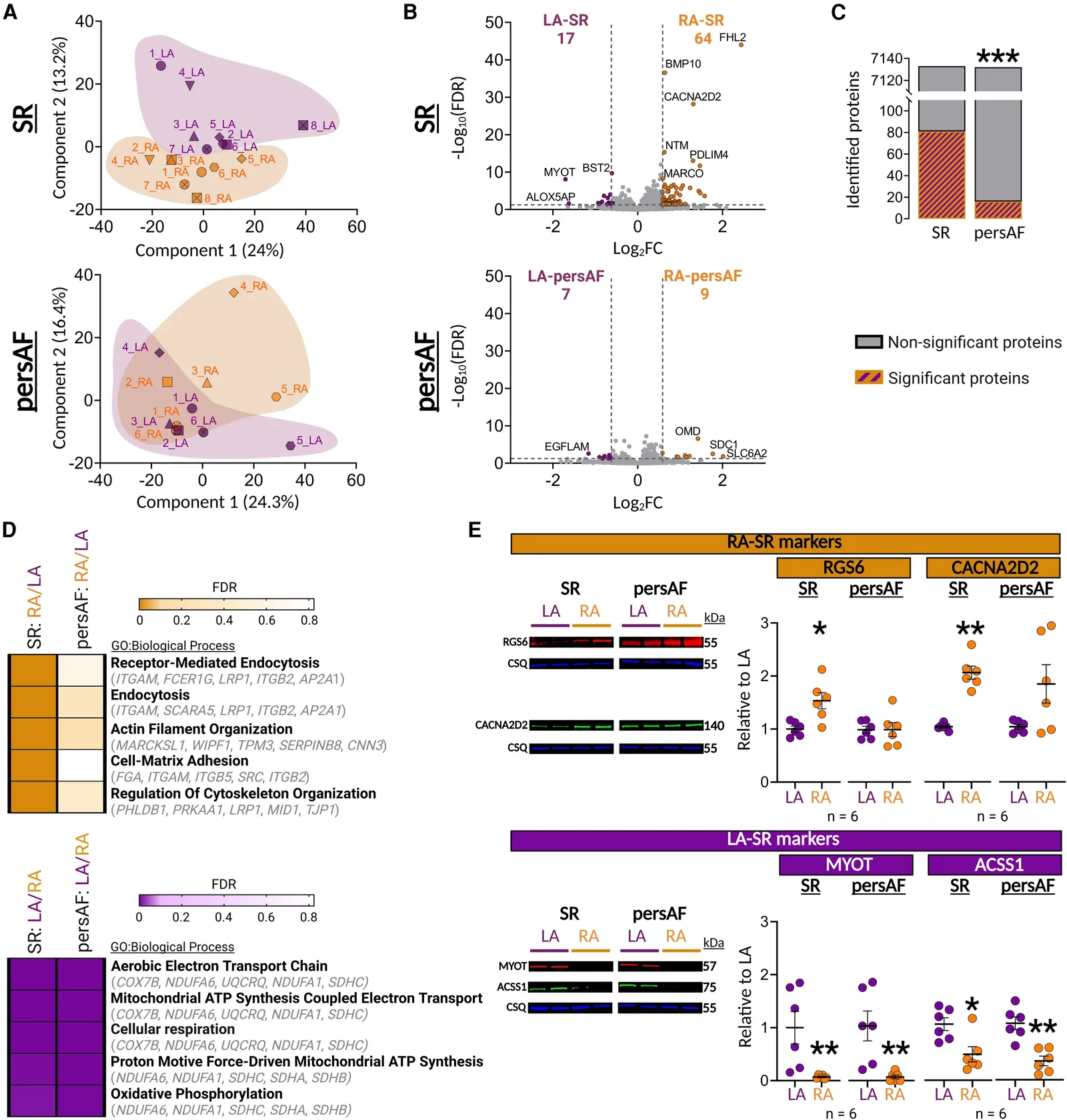
Comparative analysis of the global proteomic profiles of the RA- and LA-persAF. (*A*) PCA of RA and LA in SR (top) and persAF (bottom). Shown are the first two principal components. (*B*) Volcano plots showing the distribution of DEPs in RA vs. LA in SR (top) and persAF (bottom). Colours are assigned for the cases with FDR < 0.05 and |log_2_(FC)| > 0.58, other points are shown in grey. Legends show the total number of DEPs across both groups. (*C*) Chi-square analysis with Yates’ correction comparing the fraction of significant proteins to all identified proteins between RA vs. LA in SR and persAF. *** *P* < 0.001. (*D*) Heatmap depicting the RA-specific (left) and LA-specific (right) top 5 GO BPs in SR and their respective significance in persAF. (*E*) Immunoblots (upper left) and quantification (upper right) of RA protein markers RGS6 (regulator of G protein signaling) and CACNA2D2 (calcium voltage-gated channel auxiliary subunit α2δ2) in LA and RA in SR and persAF, normalized to CSQ (calsequestrin). Immunoblots (lower left) and quantification (lower right) of LA protein markers ACSS1 (acetyl-coenzyme A synthetase short-chain family member 1) and MYOT (myotilin) in LA and RA in SR and persAF, normalized to CSQ. * *P* < 0.05, ** *P* < 0.01 vs. LA-SR (for SR dataset) or vs. LA-persAF (for persAF dataset). Orange colour represents up-regulation in RA relative to LA and purple colour represents up-regulation in LA relative to RA. Statistical significance for proteomics data was determined using GEE analysis with Benjamini–Hochberg correction, whereas comparison of immunoblots was made using the Mann–Whitney *U* test. FC, fold change; LA, left atrium; persAF, persistent atrial fibrillation; RA, right atrium; SR, sinus rhythm. The figure was created with BioRender.com.

We next evaluated RA- and LA-persAF global proteome profiles. In SR, PCA revealed a clear separation between RA and LA samples, indicating distinct protein abundance profiles (*Figure 6A*, top, Supplementary Data  *S8*). In contrast, in persAF, RA and LA samples showed considerable overlap in PCA (*Figure 6A*, bottom), suggesting increased intra-atrial proteomic similarity.

This observation was confirmed by differential expression analysis. In SR, the number of differentially expressed proteins (DEPs) between RA and LA was significantly higher than in persAF (*Figure 6B,C*). A total of 64 proteins showed higher abundance in the RA relative to the LA and 17 in the LA relative to the RA. Among the RA-enriched proteins were canonical RA markers commonly associated with low PITX2 activity, including CACNA2D2, HCN4, RGS6, NTM, and BMP10.^13,48–52^ In contrast, LA-SR was characterized by higher abundance of structural and mitochondrial proteins, including MYOT, FLNA, and ACSS1 (see Supplementary Data  *S8*).

In persAF, the number of DEPs in RA was reduced by 85%, while LA showed a 50% reduction (*Figure 6B*, bottom). These changes were also reflected at the level of biological pathways. GO enrichment analysis (dataset pre-filtered at *P* < 0.01) identified several biological processes (BPs) that were more enriched in RA compared with LA in SR (the Supplementary Data  *S8*). The most prominent among these were related to endocytosis, cytoskeleton organization, and cell-matrix adhesion (*Figure 6D*, top), indicating robust functional specialization of the RA-SR. In contrast, the LA-SR proteome was enriched in mitochondrial proteins involved in cellular respiration, suggesting elevated energy demand (*Figure 6D*, bottom).

In persAF, RA-specific BPs were markedly diminished, in line with the reduction in RA DEPs. Conversely, LA-specific metabolic pathways remained preserved or became even more prominent. For example, the top LA-associated BP, ‘Aerobic Electron Transport Chain’ (GO:0019646), showed a highly significant enrichment in both SR (adj. *P* = 6.38 × 10^−11^) and persAF (adj. *P* = 1.03 × 10^−12^), indicating sustained mitochondrial activity in the LA.

Similar patterns are evident in the changes in RA- and LA-specific marker abundance in persAF. Consistent with the proteomics data, immunoblot analysis confirmed that the RA-specific markers RGS6 and CACNA2D2 exhibited comparable abundance between RA and LA in persAF (*Figure 6E*, top). In contrast, key LA-specific markers, MYOT and ACSS1, remained markedly up-regulated in the LA relative to the RA in persAF (*Figure 6E*, bottom).

### 3.6 RA shifts to LA proteome in persAF

To further investigate how persAF-associated remodelling contributes to the increased uniformity of the atrial chamber proteome, we analyzed the abundance of chamber-specific markers (identified in *Figure 6B*) in our larger cohort of RA samples from persAF and SR patients (described in *Figures 2–5*).

**Figure 7:**
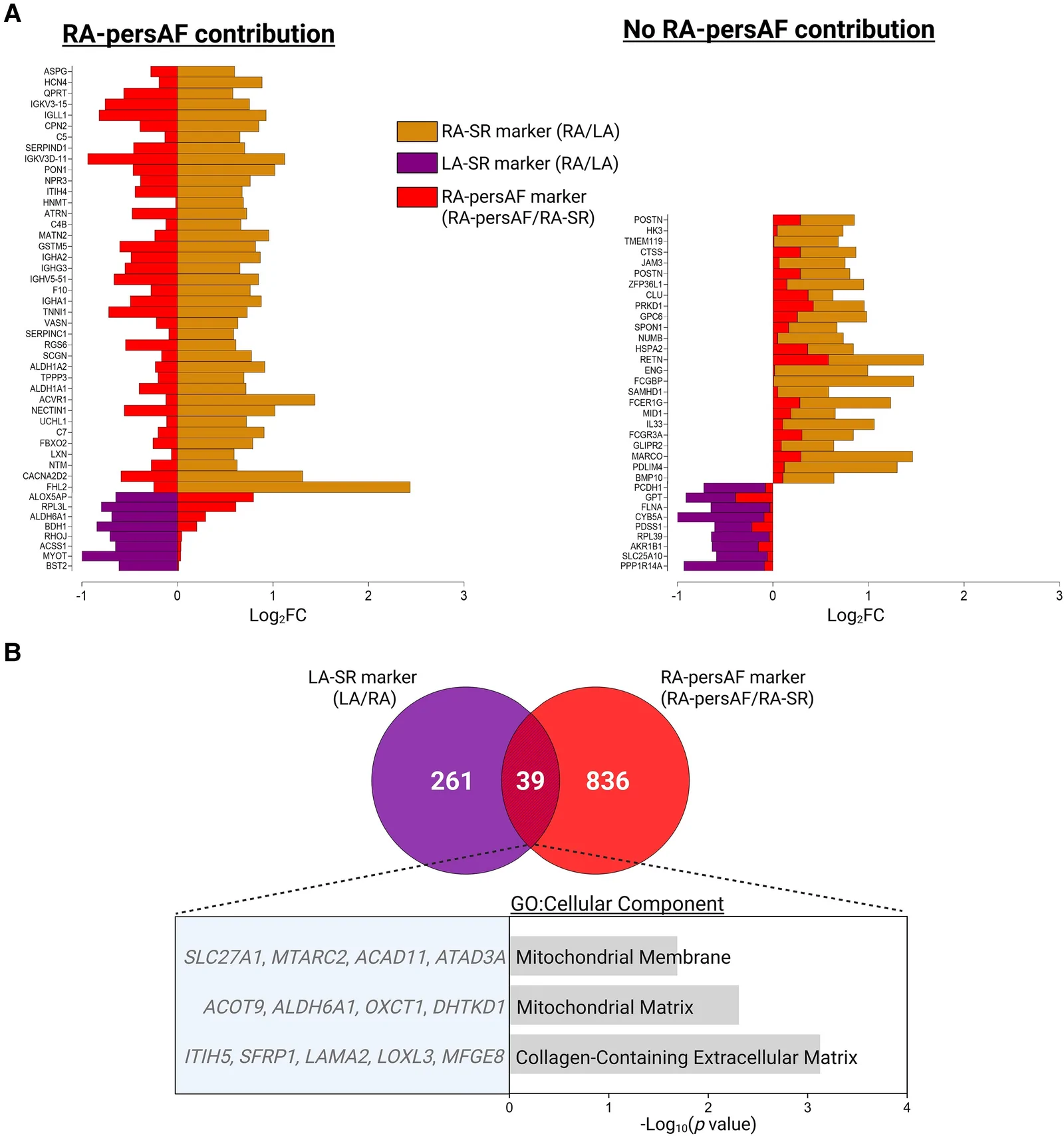
The role of the right atrium in enhanced proteome uniformity across both atria in persAF. (*A*) Mirror plots showing RA-SR markers and LA-SR markers [FDR < 0.05, |log_2_(FC)| > 0.58] and their abundance in RA-persAF relative to RA-SR. The left panel shows markers that contribute to increased atrial proteome symmetry in persAF, while the right panel shows preserved markers in RA-persAF that do not contribute to proteome symmetry. All data shown as log_2_FC. (*B*) Overlap between LA-SR (relative to RA-SR) and RA-persAF (relative to RA-SR) [*P* < 0.05, |log_2_(FC)| > 0]. The overlapping proteins were subjected to GO analysis and displayed in the lower panel together with the underlying proteins. The top terms consisting of more than 3 proteins are displayed. Statistical significance was determined using GEE analysis with Benjamini–Hochberg correction. FC, fold change; GO, gene ontology; LA, left atrium; RA, right atrium; RA-SR, right atrium from patients with sinus rhythm; RA-persAF, right atrium from patients with persistent AF.

In RA-persAF, 61% of most abundant RA-SR proteins relative to LA-SR, including HCN4, CACNA2D2, NTM and RGS6, were down-regulated (relative to RA-SR; *Figure 7A*). Conversely, 47% of most abundant LA-SR proteins (relative to paired RA-SR) were up-regulated in RA-persAF (relative to RA-SR; *Figure 7A*). This pattern indicates that RA in persAF loses part of its distinct proteomic profile while acquiring LA proteome profile, thereby potentially contributing to greater similarity between chambers.

To identify LA-enriched processes that might be more represented in RA-persAF, we selected proteins with positive log_2_FC in both LA-SR (relative to paired RA-SR) and RA-persAF (relative to RA-SR) at *P* < 0.05. The overlapping set (see Suppl. Data  *S9*) was subjected to GO analysis (*Figure 7B*), revealing enrichment of proteins predominantly localized to the mitochondrial matrix (GO:0005759) and mitochondrial membrane (GO:0031966). Notably, two key mitochondrial proteins MT-CO1 and MT-CYB were significantly down-regulated in RA-persAF (relative to RA-SR). However, they were not among the proteins enriched in LA-SR relative to RA-SR.

Overall, these results indicate that RA-persAF exhibits increased abundance of a subset of LA-SR enriched mitochondrial proteins, particularly those involved in fatty acid uptake (SLC27A1, ACAD11, ACOT9), energy metabolism (OXCT1, ALDH6A1, DHTKD1), and mitochondrial maintenance (ATAD3A, MTARC2) which may contribute to the observed atrial proteome unification.

## 4. Discussion

In this study, we investigated the AF-associated remodelling in RA relative to LA in persAF through a proteomic approach. To streamline the process and enhance the reproducibility of the analysis, we established a reference spectral library generated based on 5 anatomical regions from 5 independent donor hearts. The library was employed to analyze RA proteome of patients with SR and persAF by DIA-MS, uncovering key disease signatures and biomarkers associated with persAF. Subsequently, library-based DIA-MS facilitated the examination of both RA and LA proteomes from the same patient with SR or persAF, revealing a notable decrease in RA-specific markers and an increase in LA-specific markers within the RA, contributing to atrial chamber proteome homogenization in persAF.

### 4.1 RA remodelling in persAF

AF is a self-promoting progressive disease where structural and electrical changes induced by AF itself further promote AF initiation, maintenance and progression.^53^ We assessed the signature of persAF-associated remodelling in RA proteome.

The RA proteome in persAF exhibits remodelling consistent with well-established markers previously implicated in AF, following the same directionality. Specifically, MFG-E8 was shown to be up-regulated while GSTM2 was down-regulated at the protein level in LA appendages (LAA) of the same patient cohort.^54^ CES2 was also shown to be up-regulated in human LAA with AF, albeit at the gene transcript level.^55^ CCDC80,^56^ GABARAPL1^57^ were identified as positive blood biomarkers for AF in humans. Finally, GSTZ1 has been linked to increased AF susceptibility in a canine model and was found to be up-regulated at both the gene and protein levels in atrial tissue.^58^

PersAF-associated remodelling in the RA proteome reflects previously identified and well-established pathways that contribute to AF maintenance, including dysregulated protein homeostasis and increased ECM deposition. Additionally, and in line with previous findings, our data provide evidence of changes in protein abundance related to oxidant metabolism in persAF.

Increased protein production in the RA of patients with persAF is indicated by clusters of ribosomal and ER-associated proteins, along with elevated levels of Golgi-associated proteins. This may potentially contribute to the higher ratio of overall up-regulated vs. down-regulated proteins observed in persAF. We also detected increased levels of heat shock proteins (HSPA13, HSPA4L, and HSPE1), which assist in protein folding and protection. Similar trends were reported in the LA of persAF patients by Doll *et al*,^38^ suggesting that RA may undergo comparable remodelling in persAF.

The elevated protein load may be partially attributable to increased ECM production by activated myofibroblasts in response to mechanical stretch and inflammation.^59–63^ Elevated MMP15 in our dataset levels suggest simultaneously enhanced ECM remodelling in persAF. Fibrosis can lead to heterogeneous conduction and stabilize re-entrant drivers promoting AF.^64,65^

A strong positive correlation between lysosomal bodies and severe myofibril loss has been observed in atrial cardiomyocytes in advanced stages of AF.^66^ Myofibril degradation has also been reported in other studies involving the LA of persAF patients and in animal models.^31,54,67^ Similarly, our study identified the loss of cardiac troponins T, I, and C, tropomyosins 1 and 2, some of MYL and MYH isoforms, and MYLK in RA with persAF. The degradation of cardiac troponin C has been recently shown to contribute to impaired contractile function and to diminished cytosolic Ca^2+^ buffering that further promotes AF.^22^

Finally, the potential similarity between RA-persAF and failing ventricle appears to represent an important component of AF-associated remodelling. In contrast to transcriptomic studies,^42^ our findings did not reveal prominent commonalities between RA in persAF and healthy ventricle, but rather similarities with failing ventricle at the protein level. These included elevated levels of natriuretic peptides (in RA tissue and blood plasma) and enrichment of proteins linked to fibrosis, hypoxia, myolysis, energy metabolism and cytoskeletal organization.^43^ This overlap does not indicate a metabolic reversion to the fetal phenotype, which is a hallmark of advanced HF, as no shift from fatty acid oxidation to glycolysis was observed. Rather, this HF-like signature likely reflects stress-related adaptive processes occurring in the atria during persAF. It is important to highlight that the persAF patient cohort in this study had preserved left ventricular ejection fraction (see Supplementary material online, *Table S3*), supporting the notion that HF-associated features in the atria develop independently of ventricular dysfunction.

### 4.2 Determinants of RA-LA proteome symmetry in persAF

We subsequently investigated how AF-associated proteome remodelling compares to LA. To address this, we employed paired RA and LA samples obtained from the same patient which revealed distinct differences between SR atrial chambers. While this disparity might be partially attributed to the differences in cell type contribution between RA appendage (RAA) and LAA,^68^ we observed canonical differences between the RA and LA. The sidedness of the atria is known to be largely governed by the transcription factor PITX2, with higher activity observed in the LA.^14^ PITX2-controlled genes likely define chamber functionality and, thus, are key markers distinguishing the RA from the LA, which were prominently featured in our proteome dataset.^49,69,70^

However, we observed a prominent overlap between RA and LA samples in persAF, suggesting enhanced atrial proteome symmetry. This pattern was further supported by comparable levels of fibrosis observed in both atria, along with our previously identified AF-associated remodelling features, including myolysis, fibrosis, and failing ventricle-associated markers, which were also expressed at similar levels in both atria in persAF. These findings align with previous reports demonstrating the loss of consistent LA-RA electrophysiological gradient in patients with persAF compared with pAF or SR.^7,12^ The absence of LA-RA gradient has been largely attributed to a more uniform distribution of dominant frequencies (DF) across both atria,^8^ with the rate of DF increase also being comparable between chambers.^7,71,72^ This is further supported by unification of key ion current densities between the RA and LA exclusively in persAF^12,71^ which are key drivers of DF^71^ and, thus, AF. These findings are also in line with the unified PITX2 abundance between both chambers.^52,73^

Our data suggest two major reasons for atrial proteome unification: (ⅰ) RA exhibits a shift toward a LA proteomic profile, while the LA remains relatively stable, and (ⅱ) both atria acquire a disease-associated proteome profile to a comparable extent. More extensive RA remodelling is in line with previous transcriptome studies.^74^ On one hand, considering that AF-associated remodelling is known to promote ectopy and re-entry,^75^ it may be speculated that this may contribute to increased incidence of high-frequency sources in the RA-persAF.^76–78^ On the other hand, a comparable proteome between the two atrial chambers in persAF potentially suggests that both chambers may serve as equally vulnerable substrates promoting AF progression in the presence of widespread AF triggers.

### 4.3 Potential limitations

Although we were able to identify key proteins and processes differentially expressed between the compared groups (in substantial agreement with prior findings), our study relies on the RA and LA appendages, which may not fully represent RA and LA.

In addition, the number of paired RA and LA samples in this study is limited. Obtaining paired samples is exceptionally challenging, particularly in persAF patients without ventricular dysfunction or other comorbidities, such as mitral or coronary valve disease. While the prevalence of these conditions did not differ significantly between patient cohorts, potential confounding effects cannot be fully excluded. Paired healthy control tissues are also exceedingly rare and most published studies rely on controls with valve disease or other cardiac pathologies.

Although re-analysis of the Li *et al*.^47^ dataset markedly increased the number of detected proteins, the comparison with our persAF dataset may be influenced by technical differences in data acquisition and processing, in addition to variations in proteome coverage (6875 vs. 4456). In addition, the contribution of underlying genetic variants within the DCM cohort cannot be excluded and may introduce confounding effects, thereby precluding direct equivalence between conditions. Accordingly, the observed overlaps should be interpreted with caution.

We would like to acknowledge that the whole tissue extract provides a global disease signature but lacks cell type-specific markers and mechanisms. This approach may also mask the abundance of less abundant proteins, such as transcription factors (PITX) or ion channels (KCNQ1, KCNE1, KCNE2, KCNA5, KCNH2, KCNJ2 or KCNN3), and the latter could benefit from membrane fractionation prior to MS analysis. Nevertheless, using whole tissue extract, we were able to detect diminished levels of regulatory and auxiliary subunits of voltage-gated calcium channels, specifically CACNB2 and CACNA2D2 in RA-persAF (*Figure 2*). Moreover, it is important to note that many ion channels and Ca^2+^ handling proteins exhibit altered activity in AF primarily due to enhanced phosphorylation rather than changes in abundance levels. However, key ion currents and the abundance of relevant channels in persAF have already been extensively investigated in previous studies.^12^

### 4.4 Conclusions and potential significance

Human atrial tissue samples from AF patients are an invaluable resource that facilitated the establishment of the classical molecular mechanisms associated with AF. The RAA is one of the most commonly used patient-derived cardiac tissue sample for AF studies, primarily due to its easier accessibility as the removal of the RAA during open-heart surgery is often performed routinely in clinical practice, unlike the LAA. However, the presence of key AF triggers in the PV,^79^ along with major comorbidities primarily impacting the LA have resulted in focusing of treatment approaches predominantly to the LA. However, our findings indicate that the RA proteome exhibits the hallmark characteristics of persAF at comparable levels to LA, reflecting a global atrial unification and reinforcing the view that persAF is a biatrial disease.

Most of rate and rhythm control strategies target both, the RA and LA simultaneously (antiarrhythmic drugs, AV node ablation, cardioversion). However, catheter ablation—first-line antiarrhythmic therapy for selected patients—is predominantly used to ablate the PV in the LA.^1,2^ A high AF recurrence after ablation fostered clinicians to investigate the non-PV triggers. To date, one of the most common non-PV triggers are identified in superior vena cava and coronary sinus in the RA.^76–78^ Due to lack of large randomized studies, variability in non-PV triggers (ranging from 5 to 69%) and varying definitions of non-PV triggers in between studies, determining the benefit of targeting non-PV triggers remains challenging. However, smaller studies have demonstrated improved outcomes when pre-identified non-PV triggers, including superior vena cava and coronary sinus, are additionally ablated alongside PV.^78,80^ Furthermore, the incidence of non-PV foci has been shown to positively correlate with AF progression, with rates increasing from 2.9% in pAF to 19.1% in long-standing persAF, indicating their evolution over time if left untreated.^81^

Translational perspective Management of persistent atrial fibrillation (persAF) remains challenging and often accompanied by high recurrence rates. A deeper understanding of the mechanisms underlying AF maintenance is therefore essential to refine antiarrhythmic strategies. While left atrium is the traditional therapeutic target due to its central role in AF initiation, our findings suggest that persistent AF is also associated with major changes in the right atrium. Key AF-associated remodelling features, including fibrosis, myolysis, metabolic alterations, and failing ventricle–like characteristics were reflected at comparable levels in right and left atrial proteomes. These observations suggest that right atrium warrants greater attention in clinical management.

## Supplementary Material

cvag076_Supplementary_Data
