# Supplementary material for: Mass spectrometric proteome profiling using a deep spectral library reveals homogenization of right and left atrial proteomes in persistent atrial fibrillation patients
Source: Cardiovasc Res. 2026 Apr 2;122(8):1037–54. doi: 10.1093/cvr/cvag076 (PMC13261532; doi:10.1093/cvr/cvag076)
Supplement: cvag076_Supplementary_Data [file cvag076_Supplementary_Data.zip › Supplementary Material (10).docx]

**Mass Spectrometric Proteome Profiling Using a Deep Spectral Library Reveals Homogenization of Right and Left Atrial Proteomes in Persistent Atrial Fibrillation Patients**

Aiste Liutkute,^1,2,3*^ Takiy-Eddine Berrandou,^4*^ Stefanie Kestel,^1,2^ Moritz Schnelle,^5^ Olga Dschun,^6^ Hugo Alejandro Amedei,^5^ Lisa Neuenroth,^5,7^ Eric Rytkin,^8^ Oksana Kyshynska,^9^ George Kensah,^9^ Aschraf El-Essawi,^9^ Ahmad Fawad Jebran,^9^ Bernhard C. Danner,^9^ Hassina Baraki,^9^ Ingo Kutschka,^9^ Felix Bremmer,^6^ Henning Urlaub,^5,7^ Constanze Schmidt,^2,10,11,12^ Nabila Bouatia-Naji,^4^ Igor R. Efimov,^8,13,14^ Bianca J. J. M. Brundel,^15^ Christof Lenz,^2,3,5,7#^ Niels Voigt,^1,2,3#^

^1^Institute of Pharmacology and Toxicology, University Medical Center Göttingen, Georg-August-University Göttingen, Germany

^2^DZHK (German Center for Cardiovascular Research), Partner Site Lower Saxony, Germany

^3^Cluster of Excellence "Multiscale Bioimaging: From Molecular Machines to Networks of Excitable Cells" (MBExC), Georg-August-University Göttingen, Germany

^4^PARCC (Paris Cardiovascular Research Center), INSERM, University of Paris, Paris, France

^5^Department of Clinical Chemistry, University Medical Center, Göttingen, Germany

^6^Institute of Pathology, University Medical Center, Göttingen, Germany

^7^Bioanalytical Mass Spectrometry Group, Max Planck Institute for Multidisciplinary Sciences, Göttingen, Germany

^8^Department of Biomedical Engineering, Northwestern University, Chicago, IL, USA

^9^Department of Thoracic and Cardiovascular Surgery, University Medical Center Göttingen, Georg-August University Göttingen, Germany

^10^Department of Cardiology, University Hospital Heidelberg, Germany

^11^German Center for Cardiovascular Research Partner Site Heidelberg/Mannheim, Heidelberg University, Germany

^12^Department of Cardiology and Pneumology, University Medical Center Göttingen, Germany

^13^Department of Biomedical Engineering, The George Washington University, Washington, DC, USA

^14^Department of Medicine, Northwestern University, Chicago, IL, USA

^15^Department of Physiology, Amsterdam University Medical Center, Vrije Universiteit Amsterdam, The Netherlands

**Running title:** Proteome profiling of atria from patients with persistent atrial fibrillation

**^*^The first two authors contributed equally to this study.**

**^#^Co-corresponding Authors:**

Niels Voigt, Institute of Pharmacology and Toxicology, University Medical Center Göttingen, Robert-Koch-Straße 40, 37075 Göttingen; Phone: 0049-551-39-65174, Fax: 0049-551-39-65169; E-mail: [niels.voigt@med.uni-goettingen.de](mailto:niels.voigt@med.uni-goettingen.de)

Christof Lenz, Department of Clinical Chemistry, University Medical Center Göttingen, Robert-Koch-Straße 40, 37075 Göttingen; Phone: 0049-551-39-65192, Fax: 0049-551-39-65169; Bioanalytical Mass Spectrometry Group, Max Planck Institute for Multidisciplinary Sciences, Am Fassberg 11, 37077 Göttingen; Phone: 0049-551-39-12501; E-mail: [christof.lenz@med.uni-goettingen.de](mailto:christof.lenz@med.uni-goettingen.de)

# Table of Contents

[Table of Contents 2](#_Toc218605955)

[Supplementary Methods 3](#_Toc218605956)

[Data Analysis 3](#_Toc218605957)

[Outlier Identification 3](#_Toc218605958)

[Differential Protein Expression Analysis 3](#_Toc218605959)

[Overlap with GWAS 4](#_Toc218605960)

[Protein Mass Calculation and Subcellular Atlas Generation 4](#_Toc218605961)

[Principal Component Analysis 5](#_Toc218605962)

[Volcano Plot Analysis 5](#_Toc218605963)

[Enrichment Analysis 5](#_Toc218605964)

[Protein Association Networks 5](#_Toc218605965)

[LV-HF (DCM) Dataset Reprocessing 6](#_Toc218605966)

[Biochemical Studies 6](#_Toc218605967)

[Supplemental Tables 8](#_Toc218605968)

[Supplemental Results 16](#_Toc218605969)

[Generation of a Deep Spectral Library of the Human Cardiac Proteome 16](#_Toc218605970)

[Differential Proteome Profiling of Human Cardiac Biopsies 16](#_Toc218605971)

[Supplemental Figures 18](#_Toc218605972)

[References 24](#_Toc218605973)

# Supplementary Methods

## Data Analysis

### Outlier Identification

As a first filter, we assessed the technical fitness of each injection. Injections which had less than 50% of the highest protein groups count were excluded from further analysis to avoid normalization and imputation artifacts. In addition, outlier samples were identified by the Principal Component Analysis (PCA) and hierarchical clustering (data not shown).

### Differential Protein Expression Analysis

To analyze protein abundance, the Generalized Estimating Equations (GEE) model was implemented with the “geepack” R package^1,2^. The study involved three comparisons: 1) right atria (RA) from patients with sinus rhythm (RA-SR) vs. RA from patients with persistent atrial fibrillation (RA-persAF): This comparison included 15 individuals with replicates for RA-SR and 16 individuals with replicates for RA-persAF, except for one individual who had only one replicate. 2) RA-SR vs. left atria (LA) from patients with sinus rhythm (LA-SR): This comparison involved 8 individuals, each with 3 replicates for both RA and LA. 3) RA-persAF vs. LA-persAF: This comparison comprised 6 individuals, each with 2 replicates for both RA and LA.

The abundance values of all proteins were log_2_-transformed prior to analysis to stabilize variance and normalize the distribution of the data.

Before performing the GEE analysis, the correlation between technical replicates was assessed. The correlation coefficients were high (close to 1 for most individuals), indicating strong within-sample consistency and minimal measurement variability. Given the high correlation, the GEE model was deemed suitable as it can handle correlated measurements within subjects and estimate population-averaged effects.

For each protein, the GEE model was applied to estimate the average effects of protein abundance differences between the comparison groups. The model included the group variable as a fixed effect and accounted for within-subject correlations using an exchangeable correlation structure. We extracted coefficients and *p* values for each protein from the GEE model.

To control for multiple comparisons, we applied the FDR adjustment to the p-values using the Benjamini-Hochberg procedure^3^ via the “p.adjust” function from the “stats” package in R. Significant proteins were identified based on FDR-adjusted *p* values, with a significance threshold set at false discovery rate (FDR)-adjusted *p* < 0.05 unless otherwise stated (Suppl. Data 2 and 8, Table S1).

### Overlap with GWAS

We used the NHGRI-EBI Catalog of human genome-wide association studies (GWAS) dataset including 971 associations from 49 studies labeled with the trait 'atrial fibrillation' (EFO_0000275). The mapped genes (*p* < 5.0E-8) were compared with the RA-persAF markers (*p* < 0.05) from our dataset (Suppl. Data 3, Table S1). The genes with the same log_2_FC directionality (up- or down-regulation) across both datasets were plotted for further representation.

### Protein Mass Calculation and Subcellular Atlas Generation

The label-free quantification (LFQ) values were log_2_-transformed and technical replicates were averaged. For subsequent analyses, the leading canonical gene IDs and their associated molecular weight (MW) were utilized. Missing MW were manually assigned by averaging available MW provided by UniProt. Entries with MW less than 100 Da as well as those categorized under the GO term "blood microparticle" were excluded from the analysis (Suppl. Data 6, Table S1).

Protein mass (pg) was recalculated from the estimated protein concentration (nM) which was derived from LFQ intensity using the proteomic ruler plug-in^4^ 1.0.0.0 for Perseus. The parameters chosen were the scaling mode “Histone proteomic ruler” with ploidy of "2" and a total cellular protein concentration of 200 g/L. The list of histones identified by the software is available at Suppl. Data 6, Table S1.

A subcellular localization table was compiled by consolidating subcellular localization data from the Human Protein Atlas (v.23.0), publicly available data from published studies^5–7^ (based on leading gene ID), and manual annotations from UniProt (based on leading protein group). This resulted in 97% of proteins being assigned to the respective compartment. This enabled the calculation of protein mass for each subcellular compartment and its percentage contribution to the total protein mass (Suppl. Data 6, Table S1).

### Principal Component Analysis

PCA was conducted using Perseus v.1.6.15.0^8^. The raw data was log_2_-transformed and technical replicates were averaged to obtain mean value per biological sample which were subjected to PCA analysis proteins.

### Volcano Plot Analysis

The log_2_FC and FDR-adjusted *p* values obtained via the GEE model were used to generate volcano plots. Proteins with log_2_FC ≥ ±0.58 and FDR < 0.05 (corresponding to -log_10_FDR > 1.3) were deemed statistically significant and subsequently categorized by color.

### Enrichment Analysis

The enrichment analysis was performed using web-based tool Enrichr^9^. The list of significant proteins of interest (based on nominal *p* values, significance thresholds are indicated individually) was queried for the relevant pathways including Gene ontology (GO), Kyoto Encyclopedia of Genes and Genomes (KEGG)^10^ and Molecular Signature Database (MSigDB) Hallmark 2020^11^. The adjusted *p* values < 0.05 of biological processes and pathways were considered statistically significant.

### Protein Association Networks

To illustrate enriched protein networks underlying respective biological pathways in RA-persAF, we combined KEGG pathway analysis in conjunction with the Cytoscape software^12^. The RA-persAF protein list was pre-filtered using a *p* value cutoff 0.01 and subjected to the KEGG analysis. The proteins from the relevant pathways (adj. *p* < 0.05) were further analysed using STRING App (v.2.0.1.)^13^ in Cytoscape (v.3.9.1.) with the full STRING network type and confidence score cutoff of 0.4. Clusters consisting of minimum of 5 nodes were identified through MCL clustering using clusterMaker2 (v.2.3.4)^14^ and reuploaded to the STRING in order to generate a map containing all clusters. The individual cluster annotatation was based on KEGG-enriched term. Node color and size were adjusted using continuous mapping function based on log_2_ FC and –log_10_ *p* values, respectively. Intensity of edges (connections) is based on STRING database given score, representing the confidence level of the protein interaction. Cluster Nr. 12, associated with "Pathways of neurodegeneration" was not visualised in Fig. 3 due to its limited relevance to cardiac tissue but is provided in Suppl. Data 4, Table S1.

### LV-HF (DCM) Dataset Reprocessing

The mass spectrometry raw data for the study by Li *et al*.^15^ describing proteome analysis of left ventricular samples of patients with dilated cardiomyopathy (DCM) and healthy donors was obtained from the PRIDE repository. To ensure methodological comparability with the RA-persAF dataset, the raw files were reprocessed using Biognosys Spectronaut software (v.20) and the UniProtKB human reference proteome database, searching against the spectral library generated in this study. Protein groups were mapped to corresponding gene identifiers to avoid ambiguity from multi-entry protein groups.

This approach yielded 4,456 unique genes, representing a 33% increase (+1,544 genes) compared to the original Li *et al*. analysis (3,146 genes), with 234 genes (5%) not detected after reprocessing. None of the absent proteins overlapped with the RA-persAF and DCM markers used in the present study.

Of the 4,456 detected genes, 4,292 overlapped with our dataset, while 161 were unique to the DCM dataset; these unique proteins did not include any known DCM markers. The reprocessed dataset was analyzed using the GEE model, on log_2_-transformed protein abundances, consistent with the statistical pipeline applied to the RA-persAF dataset. This yielded 199 differentially expressed proteins (nominal *p* < 0.05) for direct comparison with the RA-persAF proteome.

## Biochemical Studies

Protein isolation and immunoblotting were performed as described previously^16,17^. Both primary and secondary antibodies (Table S4) were diluted in 1x Roti-Block (Carl Roth) buffer and incubated with the membranes at 4°C overnight or at room temperature for 1 h, respectively. Blots were imaged using Azure Sapphire system (Azure Biosystems) and analysed using Image Studio Lite v.5.2.

Each band represents a single patient sample. The signal intensity of each target protein band was normalized to the corresponding calsequestrin band, which served as the loading control.

For RA-SR *vs* RA-persAF comparisons, CSQ-normalized values were further normalized to the mean CSQ-normalized value of the RA-SR group per gel. For comparisons involving LA-SR *vs* RA-SR or LA-persAF *vs* RA-persAF, CSQ-normalized values were normalized to the mean CSQ-normalized value of the LA-SR or LA-persAF group per gel, respectively. All uncut and unedited blot images are provided in Supplementary Material.

# Supplemental Tables

**Table S1. Description of supplementary data files**

| **File name** | **Description** |
| --- | --- |
| Supplementary Data 1 | Proteome Profiling Data |
| Supplementary Data 2 | Log_2_ FC and adj. *p* values from the GEE model for RA-persAF vs RA-SR, including volcano plot dataset |
| Supplementary Data 3 | Summary results of GWAS overlap with RA-persAF markers relative to RA-SR |
| Supplementary Data 4 | Summary results of protein association network generation and pathway enrichment in RA-persAF vs RA-SR |
| Supplementary Data 5 | MSigDB Hallmark 2020 analysis results for RA-persAF vs RA-SR |
| Supplementary Data 6 | Summary results of subcellular compartmentalization assessment in RA-AF vs RA-SR |
| Supplementary Data 7 | Summary results of RA-persAF overlap with control and failing ventricle |
| Supplementary Data 8 | Log_2_ FC and adj. *p* values from the GEE model for RA vs. LA in SR and persAF, including volcano plot, PCA, and GO:BP datasets |
| Supplementary Data 9 | Summary results of LA-persAF-specific BP assessment in RA-persAF |

**Table S2. Characteristics of patients**

|  | **Sex** | **Age, y** | **BMI, kg/m^2^** | **Cause of death** |
| --- | --- | --- | --- | --- |
| D1 | Male | 54 | 28.5 | Anoxia |
| D3 | Female | 73 | 21.9 | Stroke |
| D4 | Female | 37 | 19.0 | Anoxia |
| D5 | Female | 59 | 24.9 | Stroke |
| D6 | Male | 47 | 25.2 | Head Trauma |
| D7 | Male | 45 | 26.7 | Head Trauma |
| D8 | Male | 59 | 48.8 | Anoxia |
| D10 | Male | 29 | 30.6 | Head Trauma |

BMI, body mass index.

**Table S3. Characteristics of patients involved in proteomic analysis**

|  | **Ctrl** | **persAF** | ***p* value** |
| --- | --- | --- | --- |
|  | **(n=15)** | **(n=16)** |  |
| Sex, male/female | 12/3 | 9/7 | 0.252 |
| Age, y | 65.7 ± 2.7 | 69.8 ± 2.1 | 0.097 |
| Body mass index, kg/m^2^ | 28.6 ± 1.5 | 25.5 ± 1.2 | 0.175 |
|  |  |  |  |
| CAD, n | 12 | 7 | 0.066 |
| MVD/AVD, n | 1 | 6 | 0.083 |
| CAD+MVD/AVD, n | 2 | 3 | 1.000 |
|  |  |  |  |
| Hypertension, n | 12 | 15 | 0.224 |
| Diabetes, n | 2 | 5 | 0.390 |
| Hyperlipidemia, n | 8 | 6 | 0.715 |
|  |  |  |  |
| LVEF, % | 52.0 ± 4.3 | 51.6 ± 2.9 | 0.939 |
|  |  |  |  |
| Digitalis, n | 0 | 4 | 0.100 |
| ACE inhibitors, n | 7 | 7 | 1.000 |
| AT1 blockers, n | 2 | 4 | 0.651 |
| β-Blockers, n | 10 | 14 | 0.390 |
| Dihydropyridines, n | 5 | 2 | 0.390 |
| Diuretics, n | 2 | 5 | 0.390 |
| Nitrates, n | 1 | 0 | 1.000 |
| Lipid-lowering drugs, n | 12 | 12 | 1.000 |

ACE, angiotensin-converting enzyme; AT, angiotensin receptor; CAD, coronary artery disease; LVEF, left ventricular ejection fraction; MVD/AVD, mitral/aortic valve disease. Continuous data are expressed as mean ± SEM. Comparison was made using Student’s t-test and Fisher’s exact test for continuous and categorical data, respectively.

**Table S4. Characteristics of patients involved in immunoblot analysis**

|  | **Ctrl** | **persAF** | ***p* value** |
| --- | --- | --- | --- |
|  | **(n=28)** | **(n=24)** |  |
| Sex, male/female | 24/4 | 14/10* | 0.033 |
| Age, y | 63.3 ± 2.0 | 70.8 ± 1.5** | 0.004 |
| Body mass index, kg/m^2^ | 29.4 ± 0.9 | 28.2 ± 1.0 | 0.391 |
|  |  |  |  |
| CAD, n | 23 | 12* | 0.019 |
| MVD/AVD, n | 3 | 8 | 0.086 |
| CAD+MVD/AVD, n | 2 | 4 | 0.397 |
|  |  |  |  |
| Hypertension, n | 22 | 20 | 0.736 |
| Diabetes, n | 5 | 9 | 0.130 |
| Hyperlipidemia, n | 11 | 13 | 0.403 |
|  |  |  |  |
| LVEF, % | 52.7 ± 2.1 | 50.5 ± 3.0 | 0.527 |
|  |  |  |  |
| Digitalis, n | 0 | 4* | 0.039 |
| ACE inhibitors, n | 12 | 13 | 0.578 |
| AT1 blockers, n | 4 | 6 | 0.483 |
| β-Blockers, n | 19 | 22* | 0.046 |
| Dihydropyridines, n | 6 | 7 | 0.541 |
| Diuretics, n | 6 | 13* | 0.021 |
| Nitrates, n | 1 | 0 | 1.000 |
| Lipid-lowering drugs, n | 23 | 17 | 0.510 |

ACE, angiotensin-converting enzyme; AT, angiotensin receptor; CAD, coronary artery disease; LVEF, left ventricular ejection fraction; MVD/AVD, mitral/aortic valve disease. Continuous data are expressed as mean ± SEM. **p* < 0.05 and ***p* < 0.01. Comparison was made using Student’s t-test and Fisher’s exact test for continuous and categorical data, respectively.

**Table S5. Characteristics of patients involved in histological analysis**

|  | **Ctrl** | **persAF** | ***p* value** |
| --- | --- | --- | --- |
|  | **(n=13)** | **(n=6)** |  |
| Sex, male/female | 13/0 | 4/2 | 0.088 |
| Age, y | 62.9 ± 3.0 | 71.3 ± 2.6 | 0.094 |
| Body mass index, kg/m^2^ | 28.7 ± 1.3 | 29.8 ± 2.0 | 0.644 |
|  |  |  |  |
| CAD, n | 11 | 1* | 0.010 |
| MVD/AVD, n | 0 | 3* | 0.021 |
| CAD+MVD/AVD, n | 2 | 2 | 0.557 |
|  |  |  |  |
| Hypertension, n | 11 | 4 | 0.557 |
| Diabetes, n | 5 | 2 | 1.000 |
| Hyperlipidemia, n | 9 | 1 | 0.057 |
|  |  |  |  |
| LVEF, % | 52.1 ± 5.5 | 50.8 ± 4.1 | 0.868 |
|  |  |  |  |
| Digitalis, n | 0 | 1 | 0.316 |
| ACE inhibitors, n | 9 | 2 | 0.319 |
| AT1 blockers, n | 2 | 3 | 0.262 |
| β-Blockers, n | 9 | 4 | 1.000 |
| Dihydropyridines, n | 3 | 2 | 1.000 |
| Diuretics, n | 5 | 4 | 0.350 |
| Nitrates, n | 1 | 0 | 1.000 |
| Lipid-lowering drugs, n | 12 | 6 | 1.000 |

ACE, angiotensin-converting enzyme; AT, angiotensin receptor; CAD, coronary artery disease; LVEF, left ventricular ejection fraction; MVD/AVD, mitral/aortic valve disease. Continuous data are expressed as mean ± SEM. **p* < 0.05. Comparison was made using Student’s t-test and Fisher’s exact test for continuous and categorical data, respectively.

**Table S6. Antibodies used for immunoblot analysis**

| **Antibody** | **Concentration** | **Company** | **Identifier** |
| --- | --- | --- | --- |
| CACNA2D2 | 1:2000 | Abclonal | A10267 |
| RGS6 | 1:300 | Thermo Fisher | H00009628-B01P |
| MYOT | 1:100 | Santa Cruz | sc-393957 |
| ACSS1 | 1:2000 | Cell signaling | 37041 |
| NPPA | 1:3000 | Thermo Fisher | MA5-31578 |
| NPPB | 1:2000 | antibodies-online | ABIN7010021 |
| CES2 | 1:1000 | Santa Cruz | sc-100685 |
| MFG-E8 | 1:1000 | Santa Cruz | sc-271574 |
| GSTZ1 | 1:2000 | Proteintech | 14889-1-AP |
| SLC25A36 | 1:5000 | Proteintech | 67896-1-Ig |
| TSPAN9 | 1:1000 | Proteintech | 21983-1-AP |
| CCDC80 | 1:5000 | antibodies-online | ABIN7439956 |
| GABARAPL1 | 1:2000 | Cell Signaling | 26632T |
| CSQ | 1:1000 | Thermo Fisher | PA1-913 |
| AzureSpectra 550 goat anti-rabbit | 1:5000 | Biozym | 512158 |
| IRDye 680RD donkey anti-mouse | 1:10 000 | LI-COR | 926-68072 |
| IRDye 800CW donkey anti-rabbit | 1:10 000 | LI-COR | 926-32213 |

**Table S7. Characteristics of patients involved in blood plasma analysis**

|  | **Ctrl** | **persAF** | ***p* value** |
| --- | --- | --- | --- |
|  | **(n=23)** | **(n=17)** |  |
| Sex, male/female | 17/6 | 14/3 | 0.707 |
| Age, y | 65.6 ± 1.5 | 68.9 ± 1.7 | 0.154 |
| Body mass index, kg/m^2^ | 27.9 ± 1.5 | 29.1 ± 1.2 | 0.563 |
|  |  |  |  |
| CAD, n | 17 | 3*** | 0.001 |
| MVD/AVD, n | 4 | 7 | 0.153 |
| CAD+MVD/AVD, n | 1 | 7** | 0.006 |
|  |  |  |  |
| Hypertension, n | 20 | 16 | 0.624 |
| Diabetes, n | 5 | 3 | 0.717 |
| Hyperlipidemia, n | 7 | 8 | 0.508 |
|  |  |  |  |
| LVEF, % | 48.7 ± 2.3 | 49.0 ± 3.3 | 0.939 |
|  |  |  |  |
| Digitalis, n | 1 | 2 | 0.565 |
| ACE inhibitors, n | 7 | 5 | 1.000 |
| AT1 blockers, n | 10 | 8 | 1.000 |
| β-Blockers, n | 16 | 16 | 0.107 |
| Dihydropyridines, n | 6 | 7 | 0.496 |
| Diuretics, n | 11 | 15* | 0.017 |
| Nitrates, n | 1 | 0 | 1.000 |
| Lipid-lowering drugs, n | 17 | 13 | 1.000 |

ACE, angiotensin-converting enzyme; AT, angiotensin receptor; CAD, coronary artery disease; LVEF, left ventricular ejection fraction; MVD/AVD, mitral/aortic valve disease. Continuous data are expressed as mean ± SEM. **p* < 0.05, ***p* < 0.01 and ****p* <0.001. Comparison was made using Student’s t-test and Fisher’s exact test for continuous and categorical data, respectively.

**Table S8. Concordance of primary *vs* fully adjusted tissue-proteomics effects (persAF–SR).**

| **prot** | **β _main** | ***p*_main** | **β _sens** | ***p*_sens** | **abs_log_2_FC_FDR_main** | **abs_log_2_FC_FDR_sens** |
| --- | --- | --- | --- | --- | --- | --- |
| 238 | -0.94 | 8.9E-03 | -1.24 | 1.2E-04 | <NA> | yes |
| 498 | -1.43 | 2.4E-04 | -1.78 | 2.8E-07 | yes | yes |
| 527 | 2.53 | 7.1E-04 | 2.05 | 3.0E-02 | yes | <NA> |
| 553 | -0.93 | 1.1E-02 | -1.20 | 3.7E-04 | <NA> | yes |
| 561 | 1.51 | 7.8E-05 | 1.70 | 1.1E-07 | yes | yes |
| 658 | 1.17 | 1.7E-05 | 1.16 | 6.2E-05 | yes | yes |
| 1378 | -0.64 | 1.6E-04 | -0.63 | 1.2E-03 | yes | <NA> |
| 1475 | 1.10 | 2.5E-04 | 0.96 | 6.6E-04 | yes | yes |
| 1574 | 0.92 | 5.1E-05 | 0.78 | 7.0E-03 | yes | <NA> |
| 1683 | 1.94 | 7.3E-03 | 2.35 | 1.0E-03 | <NA> | yes |
| 1694 | 0.92 | 5.2E-04 | 0.74 | 1.3E-02 | yes | <NA> |
| 1824 | 0.67 | 1.1E-03 | 0.75 | 1.1E-04 | <NA> | yes |
| 1851 | 0.94 | 1.1E-03 | 1.16 | 1.3E-05 | <NA> | yes |
| 2127 | 0.64 | 1.6E-05 | 0.63 | 1.3E-04 | yes | yes |
| 2291 | 1.05 | 2.5E-04 | 1.09 | 1.1E-03 | yes | <NA> |
| 2358 | 1.10 | 3.0E-04 | 0.85 | 7.3E-03 | yes | <NA> |
| 2700 | -0.81 | 4.5E-02 | -1.30 | 6.1E-05 | <NA> | yes |
| 3329 | -0.66 | 3.9E-05 | -0.49 | 1.6E-04 | yes | yes |
| 3845 | 0.64 | 4.2E-04 | 0.71 | 3.6E-04 | yes | yes |
| 4177 | 0.66 | 6.6E-04 | 0.44 | 1.8E-02 | yes | <NA> |
| 4178 | -0.76 | 7.5E-04 | -0.70 | 9.2E-03 | yes | <NA> |
| 5032 | 0.71 | 3.8E-05 | 0.40 | 1.5E-02 | yes | <NA> |
| 5256 | 0.64 | 6.2E-04 | 0.64 | 2.9E-04 | yes | yes |
| 5276 | 0.59 | 2.9E-04 | 0.53 | 4.3E-03 | yes | <NA> |
| 5591 | 0.83 | 2.3E-04 | 0.59 | 4.4E-03 | yes | <NA> |
| 5609 | 0.61 | 5.8E-04 | 0.58 | 2.6E-03 | yes | <NA> |
| 5821 | 0.99 | 3.3E-03 | 1.27 | 7.2E-04 | <NA> | yes |
| 6317 | -0.58 | 1.2E-07 | -0.64 | 2.3E-07 | yes | yes |

β_main/*p*_main = primary model; β_sens/*p*_sens = fully adjusted model (Age, Sex, Drugs-PC1, CAD, MVD/AVD). abs_log_2_FC_fdr_* indicates proteins meeting FDR < 0.05 and |log_2_FC| > 0.58 in that analysis.

# Supplemental Results

***Generation of a Deep Spectral Library of the Human Cardiac Proteome***

We generated an annotated tandem mass spectrometry (MS/MS) spectral library from RA, LA, RV, LV and septum samples. To minimize sampling effects, material from three individual, randomly chosen pieces of tissue per cavity was lysed and tryptically digested separately, then pooled at the tryptic peptide level for further analysis. Each peptide pool was fractionated into 20 fractions using a staggered pooling scheme and analyzed by DDA-MS in triplicate to achieve comprehensive sampling. From the combined DDA-MS data, an annotated spectral library was generated that encompassed data on 9,159 non-redundant protein groups representing 13,539 proteins from the UniProtKB database, evidenced by 111,772 peptide sequences of which 40,537 are classified as proteotypic (Fig. S2A)^18^. Of note, peptides are not only characterized by their MS/MS data, but also by their indexed retention time (Fig. S2B)^19^ and their inverse reduced ion mobility 1/K_0_ (Fig. S2C) as a prerequisite for high-quality assignment in DIA-MS experiments. Peptide and protein identifications were transcribed into an annotated MS/MS spectral library.

## Differential Proteome Profiling of Human Cardiac Biopsies

Next, we used the reference MS/MS spectral library to extract quantitative peptide and protein information from single-shot DIA-MS. Using the library, we observed consistent detection of proteins across all injections (Figure S2D). Following quantitative normalization and outlier identification based on sample input amounts and technical fitness, we obtained a highly consistent data matrix across 121 injections representing 55 individual samples.

In the full dataset, the median number of identified protein groups was 5,984, with a standard deviation of 195 (3.3%) and a range of 5,222 to 6,303 (data not shown). When stratified by experimental groups, the values remained similarly consistent: in LA-SR samples, the median was 5,963 with a standard deviation of 263 (4.5%) and a range of 5,222 to 6,176; in LA-persAF, the median was 5,891 with a standard deviation of 122 (2.1%) and a range of 5,609 to 6,050; in RA-SR, the median was 5,868 with a standard deviation of 168 (2.9%) and a range of 5,320 to 6,303; and in RA-persAF, the median was 5,993 with a standard deviation of 110 (1.8%) and a range of 5,774 to 6,170. These values confirm robust and reproducible protein detection across both atrial chambers and rhythm conditions.

Analysis of quantitative variability between samples in each experimental group exhibited excellent reproducibility, with median in-group coefficient of variation (CV) ranging from 14.2% to 20.1% (Fig. S2E).

To evaluate whether our spectral library (generated from non-failing donor hearts) is suitable to capture persAF-specific proteins, we compared our data set to a set of 30 core persAF markers consistently identified in two independent proteomic studies^6,20^. We were able to detect all of these markers in our dataset (Fig. S2F). With regard to the extracellular matrix as a key determinant of cardiac tissue remodeling, we detected 362 *bona fide* extracellular matrix (ECM) proteins contained in the matrisome database^21^ even without decellularization, which is comparable with dedicated ECM studies^22^. We conclude that our methodological approach, while not providing complete cardiac proteome profiles, is highly suitable to study the processes underlying tissue remodeling in the context of persAF, despite the use of healthy donor tissue-based library.

# Supplemental Figures


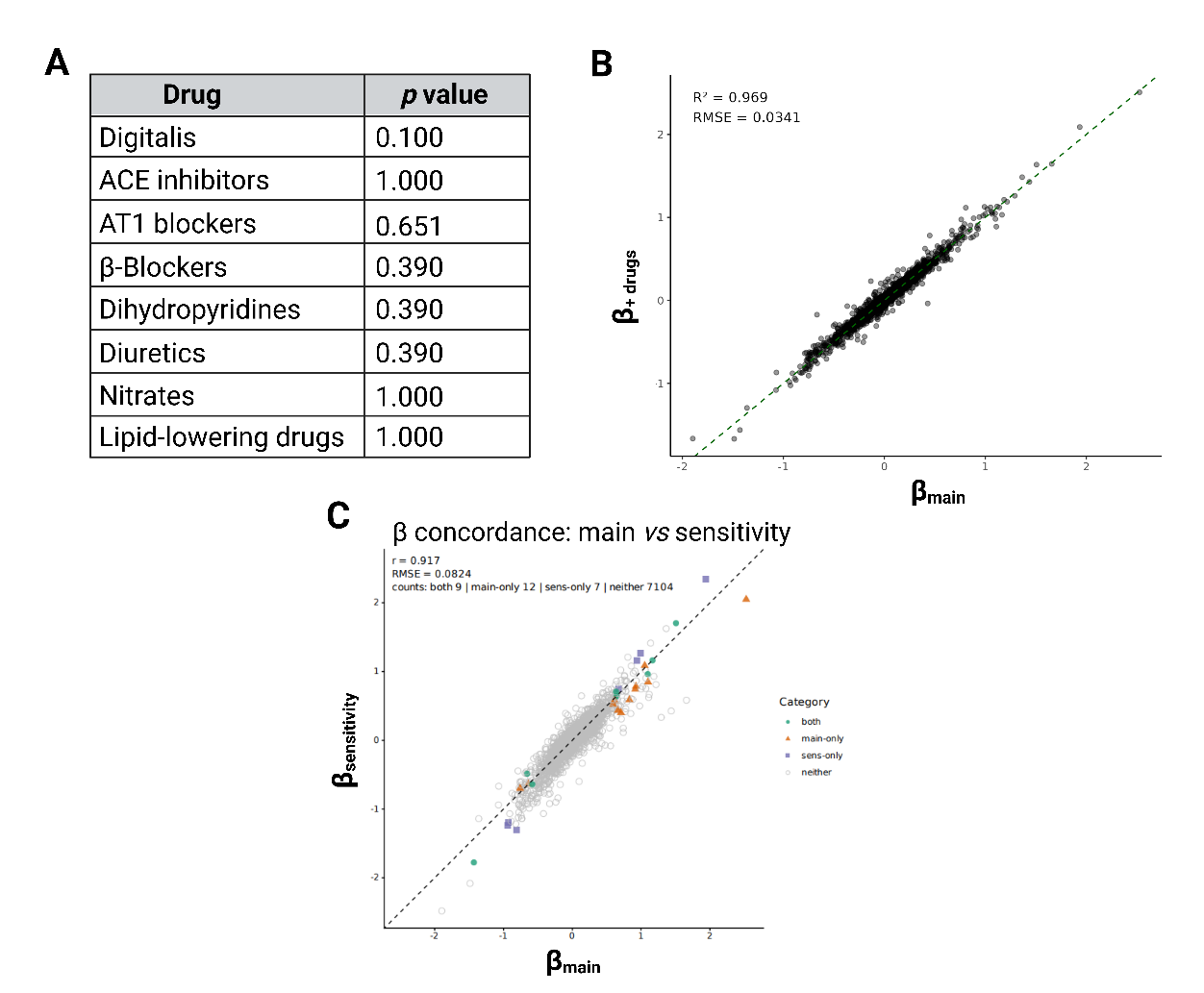


**Fig. S1 Assessment of potential confounding effect by medication use. A,** Summary of the *p* values obtained by Fisher’s exact test aimed to evaluate the effect of 8 major drug categories between RA-SR and persAF patients. **B,** Scatter plot comparing group-effect coefficients (β) before and after adjustment for overall medication burden (Drugs-PC1). The high agreement (R^2^ = 0.969; RMSE = 0.0341) indicates that medication use did not substantially influence the estimated group effects. **C,** β–β concordance between the primary model and the fully adjusted model (Age, Sex, Drugs-PC1, CAD, MVD/AVD). Agreement remained high (r = 0.917; RMSE = 0.082); counts: both significant = 9, main-only = 12, sensitivity-only = 7.


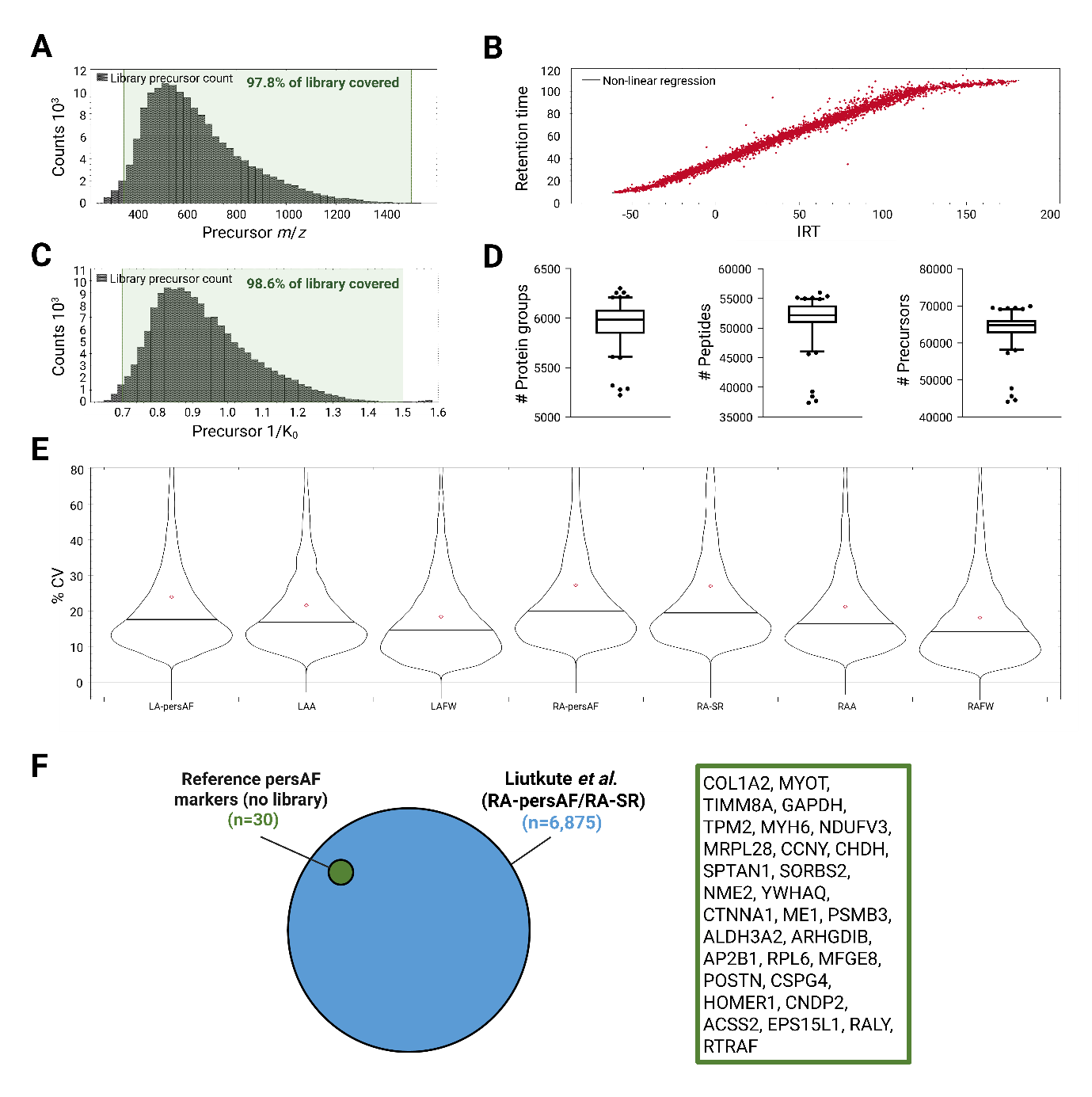


**Fig. S2 Validation of the library.** **A,** DDA MS/MS spectral library, *m*/*z* distribution of peptide precursors. Marked area represents precursor isolation of *m*/*z* 350-1400 in DIA-MS, covering 97.6% of the library. **B,** DDA MS/MS spectral library, representative plot of retention time vs IRT. **C,** DDA MS/MS spectral library, 1/K_0_ distribution of peptide precursors. Marked area represents precursor isolation of 1/K_0_ 0.7-1.5 in DIA-MS, covering 98.6% of the library. **D,** Boxplots of protein group, peptide and precursor detections across samples (n=121, 5/95% percentiles). **E,** Violin plots of protein *g* coefficients of variation, analysed by sample group. Median CV range from 14.2% to 20.1%. **F,** The schematic representation of the reference persAF protein markers identified in previous studies using library-free approach^6,20^ within the proteome dataset of this study (generated via healthy donor-based library; left). All 30 reference persAF markers (right) were detected in our dataset, supporting the suitability of the spectral library derived from non-failing hearts. CV, coefficient of variation; IRT, indexed retention time; LAapp, left atrial appendage (donors, also called LA-SR in the main text); LAFW, left atrial free wall (donors, also called LA-SR in the main text); LA-persAF, left atrium from patients with persistent AF; RAapp, right atrial appendage (donors, also called RA-SR in the main text); RAFW, right atrial free wall (donors, also called RA-SR in the main text); RA-persAF, right atrium from patients with persistent AF; RA-SR, right atrium from patients with sinus rhythm.

**
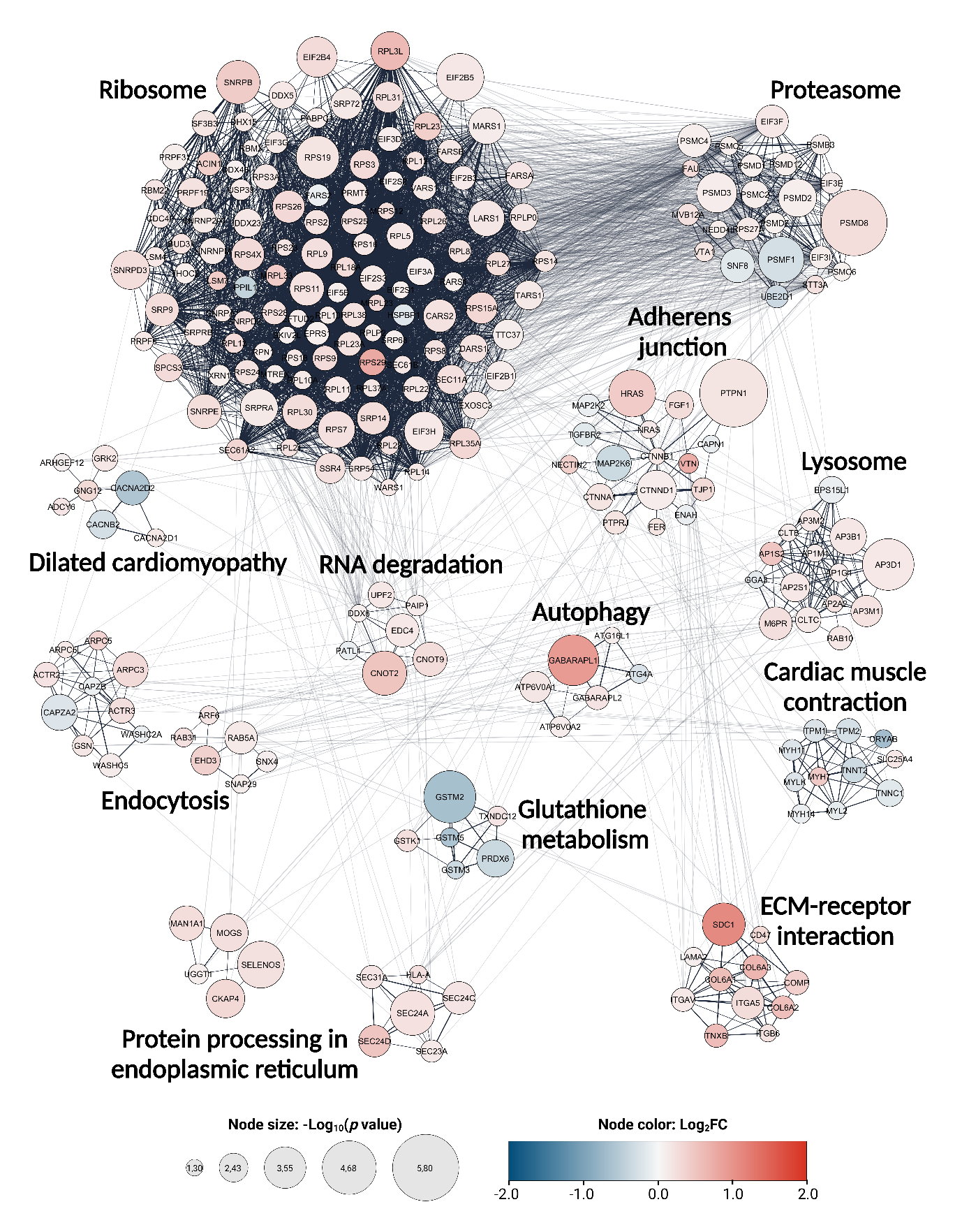
**

**Fig. S3 Protein association network with pathway enrichment in the RA-AF proteome (full size).** Each node represents a protein; node size indicates –log_10_(*p* value), node colour indicates log_2_ fold change, and edge thickness reflects interaction confidence. KEGG pathway associations were assigned to each cluster. Statistical significance was assessed using generalized estimating equations with Benjamini–Hochberg correction. ECM, extracellular matrix; FC, fold change.


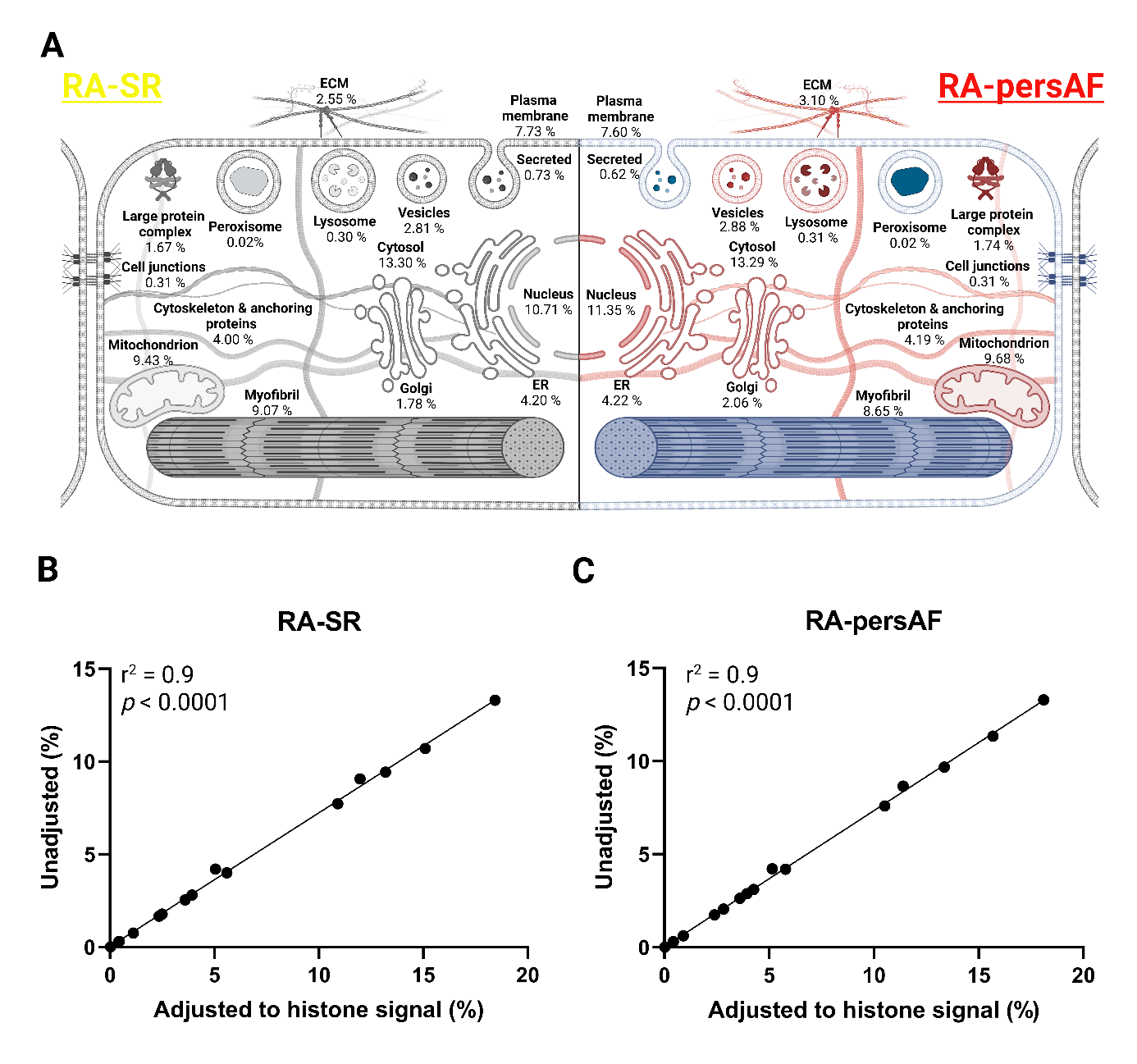


**Fig S4 Subcellular proteome model of RA-persAF (unadjusted). A,** Schematic representation of an average right atrial appendage cardiomyocyte illustrating the subcellular distribution of protein mass fractions per cell, estimated using proteomic ruler approach. Values are normalized to total cellular protein content. Left: RA-SR (control). Right: RA-persAF. In both panels, compartments are labeled with their absolute percentage contribution (%) to total protein mass per cell, enabling direct comparison between conditions. Please note that unassigned proteins were not included in the figure. Scatter plot comparing subcellular compartment protein mass fractions (%) derived from histone-adjusted proteomic ruler analysis (x-axis) and unadjusted raw LFQ-based analysis (y-axis) in RA-SR (**B**) and RA-persAF (**C**). The diagonal line indicates the line of identity (Y = X). Pearson correlation coefficients (r^2^) and *p* values are shown. ECM, extracellular matrix; ER, endoplasmic reticulum; RA-persAF, right atrium from patients with persAF; RA-SR, right atrium from patients with sinus rhythm. The figure was created with BioRender.com.


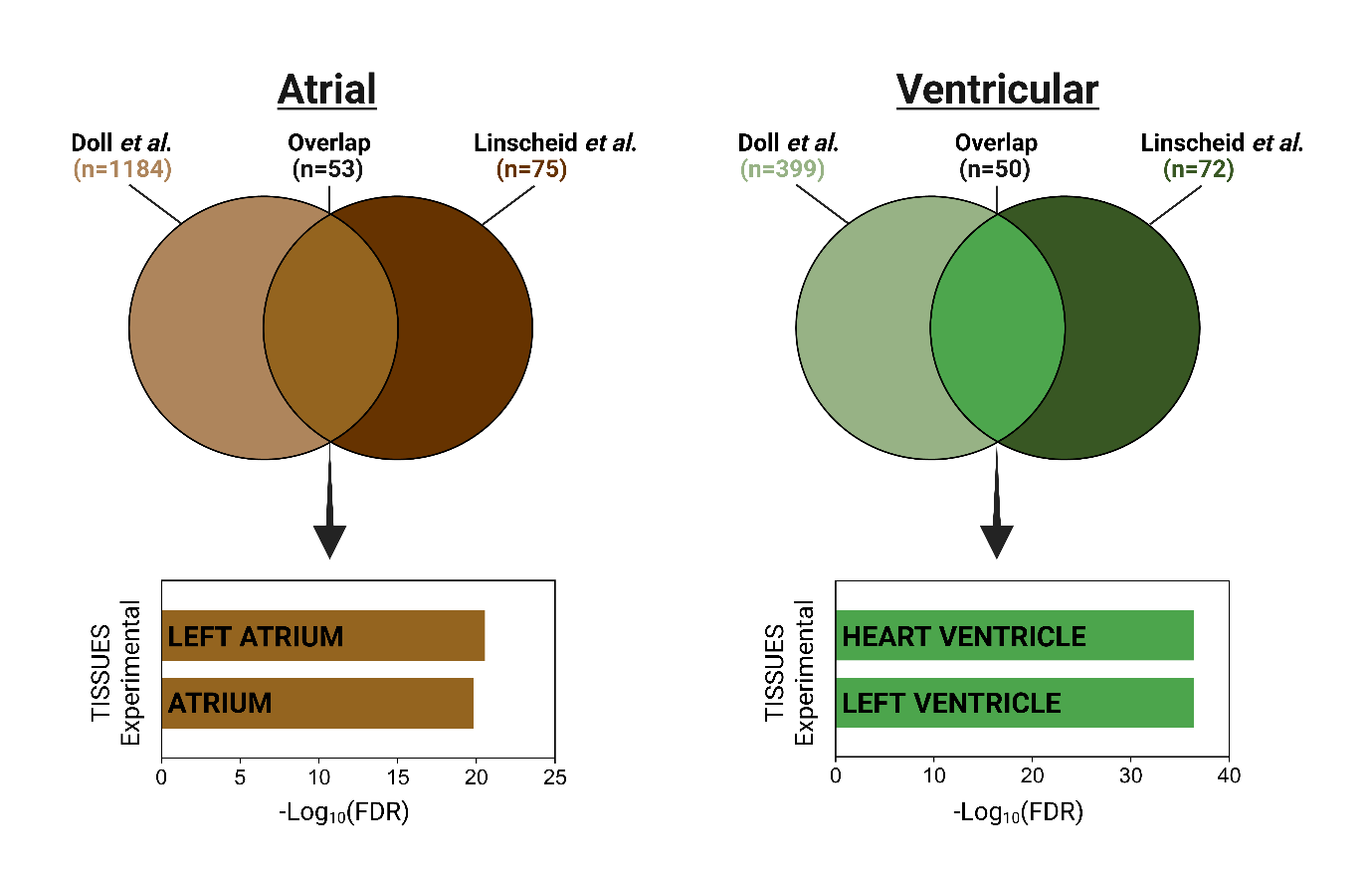


**Fig. S5 Derivation of robust human atrial and ventricular proteome marker sets.** To obtain robust human atrial and ventricular proteome markers, we employed the atrial and ventricular proteome markers available from from two independent published cardiac proteome studies^6,23^ and identified the overlapping markers (top). These overlapping proteins were further validated using the TISSUES Experimental 2025 database, which confirmed their specificity to atrial and ventricular heart chambers. FDR, false discovery rate.

# References

1. Liang KY, Zeger SL. Longitudinal data analysis using generalized linear models. *Biometrika*. 1986;73(1):13–22.

2. Halekoh U, Højsgaard S, Yan J. The R package geepack for generalized estimating equations. *J Stat Softw*. 2006;15(2):1–11.

3. Benjamini Y, Hochberg Y. Controlling the False Discovery Rate: A Practical and Powerful Approach to Multiple Testing. *J R Stat Soc Ser B Stat Methodol*. 1995;57(1):289–300.

4. Wiśniewski JR, Hein MY, Cox J, Mann M. A “proteomic ruler” for protein copy number and concentration estimation without spike-in standards. *Mol Cell Proteomics*. 2014;13(11):3497-3506.

5. Thul PJ, Akesson L, Wiking M, Mahdessian D, Geladaki A, Ait Blal H, Alm T, Asplund A, Björk L, Breckels LM, Bäckström A, Danielsson F, Fagerberg L, Fall J, Gatto L, Gnann C, Hober S, Hjelmare M, Johansson F, Lee S, Lindskog C, Mulder J, Mulvey CM, Nilsson P, Oksvold P, Rockberg J, Schutten R, Schwenk JM, Sivertsson A, Sjöstedt E, Skogs M, Stadler C, Sullivan DP, Tegel H, Winsnes C, Zhang C, Zwahlen M, Mardinoglu A, Pontén F, Von Feilitzen K, Lilley KS, Uhlén M, Lundberg E. A subcellular map of the human proteome. *Science*. 2017;356(6340):eaal332.

6. Doll S, Dreßen M, Geyer PE, Itzhak DN, Braun C, Doppler SA, Meier F, Deutsch MA, Lahm H, Lange R, Krane M, Mann M. Region and cell-type resolved quantitative proteomic map of the human heart. *Nat Commun*. 2017;8(1):1469.

7. Itzhak DN, Tyanova S, Cox J, Borner GHH. Global, quantitative and dynamic mapping of protein subcellular localization. *Elife*. 2016; 5:e16950.

8. Tyanova S, Temu T, Sinitcyn P, Carlson A, Hein MY, Geiger T, Mann M, Cox J. The Perseus computational platform for comprehensive analysis of (prote)omics data. *Nat Methods*. 2016;13(9):731–740.

9. Chen EY, Tan CM, Kou Y, Duan Q, Wang Z, Meirelles GV, Clark NR, Ma’ayan A. Enrichr: Interactive and collaborative HTML5 gene list enrichment analysis tool. BMC *Bioinformatics*. 2013;14:128.

10. Kanehisa M, Goto S. KEGG: Kyoto encyclopedia of genes and genomes. *Nucleic Acids Res*. 2000;28(1):27–30.

11. Subramanian A, Tamayo P, Mootha VK, Mukherjee S, Ebert BL, Gillette MA, Paulovich A, Pomeroy SL, Golub TR, Lander ES, Mesirov JP. Gene set enrichment analysis: A knowledge-based approach for interpreting genome-wide expression profiles. *Proc Natl Acad Sci U S A*. 2005;102(43):15545–15550.

12. Shannon P, Markiel A, Ozier O, Baliga NS, Wang JT, Ramage D, Amin N, Schwikowski B, Ideker T. Cytoscape: A software environment for integrated models of biomolecular interaction networks. Genome Res. 2003;13:2498-2504.

13. Szklarczyk D, Morris JH, Cook H, Kuhn M, Wyder S, Simonovic M, Santos A, Doncheva NT, Roth A, Bork P, Jensen LJ, Von Mering C. The STRING database in 2017: Quality-controlled protein-protein association networks, made broadly accessible. *Nucleic Acids Res*. 2017;45(D1):D362–D368.

14. Morris JH, Apeltsin L, Newman AM, Baumbach J, Wittkop T, Su G, Bader GD, Ferrin TE. ClusterMaker: A multi-algorithm clustering plugin for Cytoscape. *BMC Bioinformatics*. 2011;12:1–14.

15. Li M, Parker BL, Pearson E, Hunter B, Cao J, Koay YC, Guneratne O, James DE, Yang J, Lal S, O’Sullivan JF. Core functional nodes and sex-specific pathways in human ischaemic and dilated cardiomyopathy. *Nat Commun*. 2020;11(1):2843.

16. Voigt N, Li N, Wang Q, Wang W, Trafford AW, Abu-Taha I, Sun Q, Wieland T, Ravens U, Nattel S, Wehrens XHT, Dobrev D. Enhanced sarcoplasmic reticulum Ca^2+^ leak and increased Na^+^-Ca^2+^ exchanger function underlie delayed afterdepolarizations in patients with chronic atrial fibrillation. *Circulation*. 2012;125(17):2059–2070.

17. Fakuade FE, Hubricht D, Möller V, Sobitov I, Liutkute A, Döring Y, Seibertz F, Gerloff M, Pronto JRD, Haghighi F, Brandenburg S, Alhussini K, Ignatyeva N, Bonhoff Y, Kestel S, El-Essawi A, Jebran AF, Großmann M, Danner BC, Baraki H, Schmidt C, Sossalla S, Kutschka I, Bening C, Maack C, Linke WA, Heijman J, Lehnart SE, Kensah G, Ebert A, Mason FE, Voigt N. Impaired intracellular calcium buffering contributes to the arrhythmogenic substrate in atrial myocytes from patients with atrial fibrillation. *Circulation*. 2024;150(7):544-559.

18. Mallick P, Schirle M, Chen SS, Flory MR, Lee H, Martin D, Ranish J, Raught B, Schmitt R, Werner T, Kuster B, Aebersold R. Computational prediction of proteotypic peptides for quantitative proteomics. *Nat Biotechnol*. 2007;25(1):125-131.

19. Bruderer R, Bernhardt OM, Gandhi T, Reiter L. High-precision iRT prediction in the targeted analysis of data-independent acquisition and its impact on identification and quantitation. *Proteomics*. 2016;16(15-16):2246-2256.

20. Rennison JH, Li L, Lin CR, Lovano BS, Castel L, Wass SY, Cantlay CC, McHale M, Gillinov AM, Mehra R, Willard BB, Smith JD, Chung MK, Barnard J, Van Wagoner DR. Atrial fibrillation rhythm is associated with marked changes in metabolic and myofibrillar protein expression in left atrial appendage. *Pflugers Arch Eur J Physiol*. 2021;473(3):461-475.

21. Naba A, Clauser KR, Hoersch S, Liu H, Carr SA, Hynes RO. The matrisome: in silico definition and in vivo characterization by proteomics of normal and tumor extracellular matrices. *Mol Cell Proteomics*. 2012;11(4):M111.01464717.

22. Buck KM, Rogers HT, Gregorich ZR, Mann MW, Aballo TJ, Gao Z, Chapman EA, Perciaccante AJ, Price SJ, Lei I, Tang PC GY. Extracellular Matrix Alterations in Chronic Ischemic Cardiomyopathy Revealed by Quantitative Proteomics. 2025;*bioRxiv [Preprint]*.

23. Linscheid N, Santos A, Poulsen PC, Mills RW, Calloe K, Leurs U, Ye JZ, Stolte C, Thomsen MB, Bentzen BH, Lundegaard PR, Olesen MS, Jensen LJ, Olsen J V., Lundby A. Quantitative proteome comparison of human hearts with those of model organisms. *PLoS Biol*. 2021;19(4).
