# Supplementary figures and images for: Mass spectrometric proteome profiling using a deep spectral library reveals homogenization of right and left atrial proteomes in persistent atrial fibrillation patients
Source: Cardiovasc Res. 2026 Apr 2;122(8):1037–54. doi: 10.1093/cvr/cvag076 (PMC13261532; doi:10.1093/cvr/cvag076)

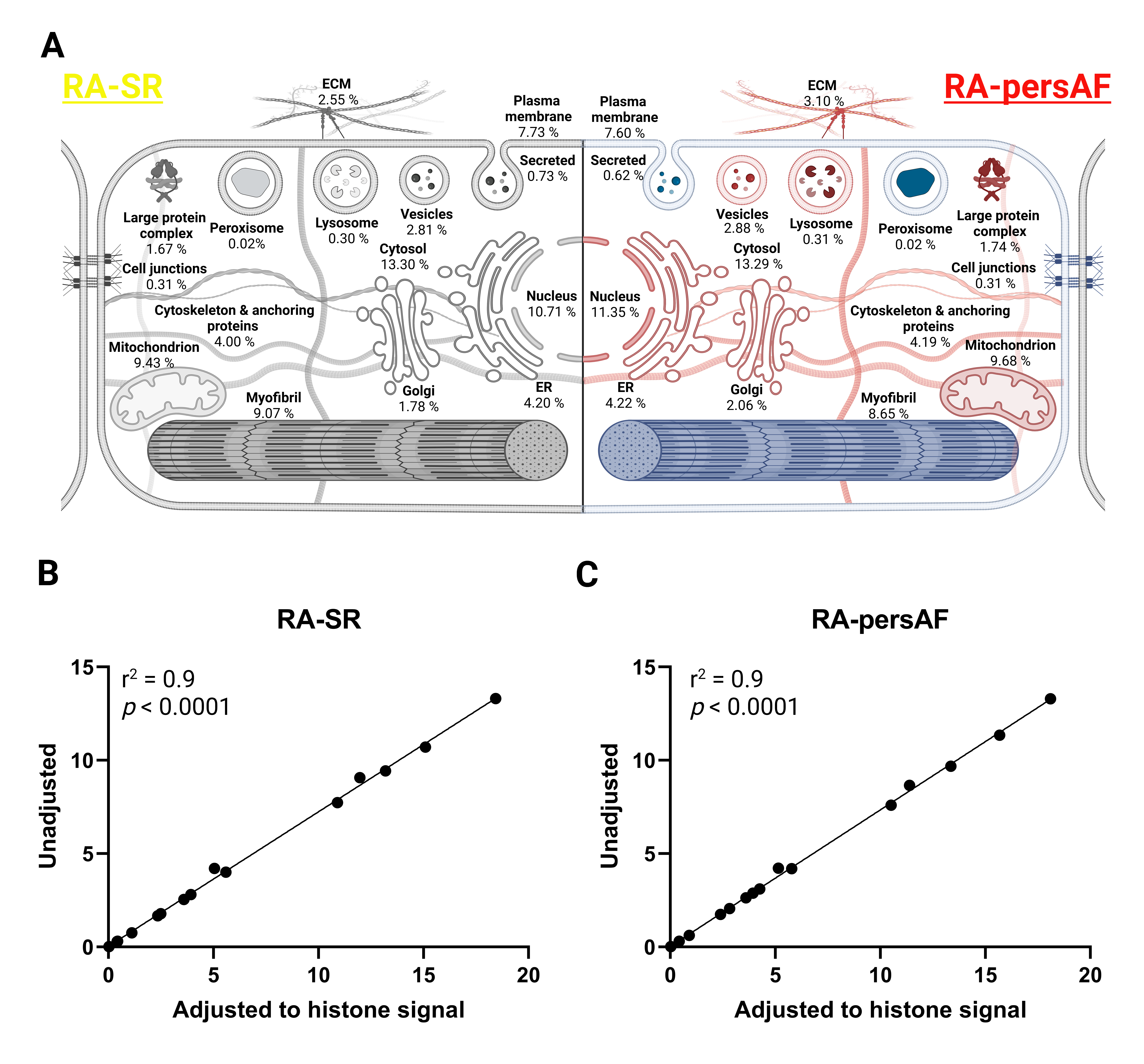

Supplement: cvag076_Supplementary_Data [file cvag076_Supplementary_Data.zip › Fig. S4.png]

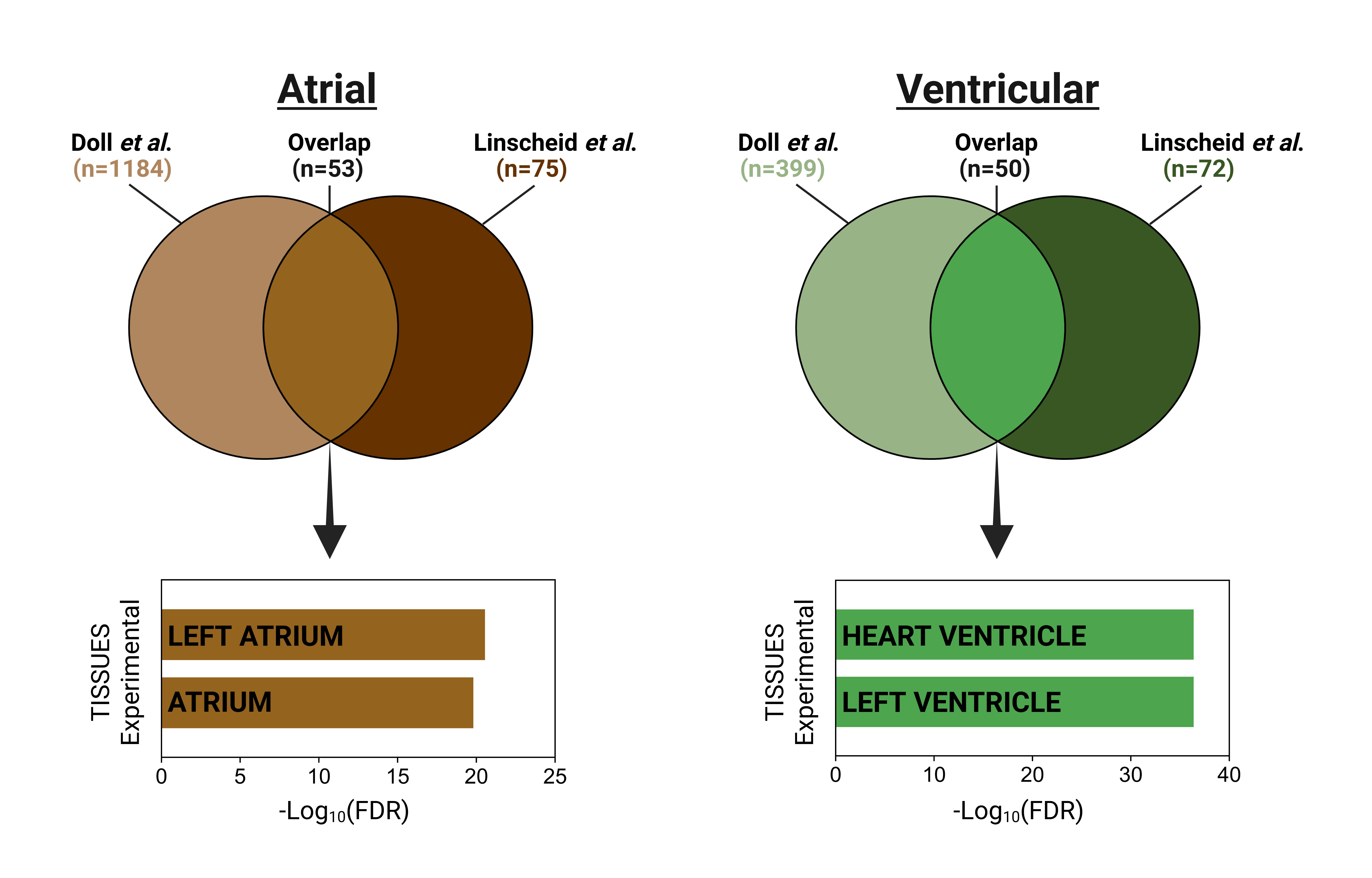

Supplement: cvag076_Supplementary_Data [file cvag076_Supplementary_Data.zip › Fig. S5.png]
